# Supplementary material for: Effectiveness of a universal digital–human parenting intervention in promoting early childhood development and protection: A pragmatic cluster randomized controlled trial
Source: PLOS Digit Health. 2026 May 7;5(5):e0001357. doi: 10.1371/journal.pdig.0001357 (PMC13152119; doi:10.1371/journal.pdig.0001357)
Supplement: S1 Text — Table 1: Detailed Description of Intervention Design and Delivery; Table 2: Outcome Measures; Table 3a. Minimum Detectable Effect Size (Beta) under Linear Model for Early Learning and Stimulation, Table 3b. Minimum Detectable Effect Size (IRR) under Poisson Model for Caregiver-Perpetrated Violence; Table 4. Demographic Characteristics and Baseline Measures of Participants by Group (T0); Table 5. Participant Engagement in the Chatbot; Fig 1. Distribution of Total Modules Completed Among Participants in the Intervention Group; Table 6. Means and Standard Deviations of Outcome Measures at Baseline, Immediate Post-Intervention; Table 7. Distribution Check for Caregiver-Perpetrated Violence; Table 8. Per-Protocol Sensitivity Analysis for Primary Outcomes; Table 9. Sensitivity Analyses Adjusting for Covariates: Intervention Effects on Primary and Secondary Outcomes; Table 10a. Subgroup and Moderation Analyses by Caregiver Disability Status, Table 10b. Subgroup and Moderation Analyses by Child Disability Status; Table 11. Changes in Outcome Variables from Baseline to Post-Intervention and 12-Month Follow-Up in the Intervention Group; Table 12. Baseline Characteristics by 12-Month Follow-Up Status in the Intervention Group; Table 13 Baseline Predictors of Compliance in the Intervention Group; Table 14. Estimated Complier Average Causal Effects on Primary and Secondary Outcome Variables; Table 15. Cluster-Level Compliance Rates in the Intervention Group; Methods. Pragmatic Trial Orientation; Reference. (DOCX) [file pdig.0001357.s002.docx]

**Supplement to:** Fang, Z., Han, Q., Ruan, R., Shi, X., Zhang, C., Ruan, D., Fang, X., Vallance, I. Lachman, J.M. (2026). Effectiveness of a universal digital–human parenting intervention in promoting early childhood development and protection: A pragmatic cluster randomized controlled trial. *PLOS Digital Health.*

**S1 Text**

Table of Contents

[Table 1. Detailed Description of Intervention Design and Delivery 2](#_Toc225499484)

[Table 2. Outcome Measures 4](#_Toc225499485)

[Table 3a. Minimum Detectable Effect Size (Beta) under Linear Model for Early Learning and Stimulation 7](#_Toc225499486)

[Table 3b. Minimum Detectable Effect Size (IRR) under Poisson Model for Caregiver-Perpetrated Violence 7](#_Toc225499487)

[Table 4. Demographic Characteristics and Baseline Measures of Participants by Group (T0) 7](#_Toc225499488)

[Table 5. Participant Engagement in the Chatbot 8](#_Toc225499489)

[Fig 1. Distribution of Total Modules Completed Among Participants in the Intervention Group 9](#_Toc225499490)

[Table 6. Means and Standard Deviations of Outcome Measures at Baseline, Immediate Post-Intervention 9](#_Toc225499491)

[Table 7. Distribution Check for Caregiver-Perpetrated Violence 10](#_Toc225499492)

[Table 8. Per-Protocol Sensitivity Analysis for Primary Outcomes 10](#_Toc225499493)

[Table 9. Sensitivity Analyses Adjusting for Covariates: Intervention Effects on Primary and Secondary Outcomes 10](#_Toc225499494)

[Table 10a. Subgroup and Moderation Analyses by Caregiver Disability Status 11](#_Toc225499495)

[Table 10b. Subgroup and Moderation Analyses by Child Disability Status 13](#_Toc225499496)

[Table 11. Changes in Outcome Variables from Baseline to Post-Intervention and 12-Month Follow-Up in the Intervention Group 14](#_Toc225499497)

[Table 12. Baseline Characteristics by 12-Month Follow-Up Status in the Intervention Group 16](#_Toc225499498)

[Table 13. Baseline Predictors of Compliance in the Intervention Group 16](#_Toc225499499)

[Table 14. Estimated Complier Average Causal Effects on Primary and Secondary Outcome Variables 17](#_Toc225499500)

[Table 15. Cluster-Level Compliance Rates in the Intervention Group 18](#_Toc225499501)

[Methods. Pragmatic Trial Orientation 18](#_Toc225499502)

[References: 19](#_Toc225499503)

## Table 1. Detailed Description of Intervention Design and Delivery

| **Component** | **Description** |
| --- | --- |
| **Name of intervention** | *Keyushiguang* (adapted from Parenting for Lifelong Health <PLH> – ParentText Young Children, a rule-based chatbot-delivered parenting intervention for caregivers of children aged 2 to 9) |
| **PLH theoretical basis** | Grounded in social learning theory and attachment theory; developed in collaboration with WHO, UNICEF, and other universities and partners; evidence-based content tested in 20+ RCTs across Asia, Africa, and Eastern Europe.[1–3] |
| **Cultural adaptation** | Jul 2023 – Feb 2024, guided by ADAPT framework:[4,5]   - Surface adaptations: linguistic translation, localization of visuals, examples, and metaphors. - Deep adaptations: content modifications to reflect local culture, e.g., filial piety, budgeting, communication norms, dietary practices. - The process involved literature and policy review, stakeholder consultations with caregivers, preschool teachers, and social workers, expert review panels, iterative content revision, and small-scale feasibility testing prior to trial implementation. - Care was taken to preserve the core behavior change principles of the original PLH intervention while enhancing contextual relevance and acceptability. |
| **Topics covered** | 1. Parent–child relationships (5 modules)  2. Child development (3 modules)  3. Child behavior management (5 modules)  4. Child learning (4 to 6 modules)  5. Proactive parenting (5 modules)  6. Child safety & nutrition (6 modules)  7. Family relationships (5 modules)  8. Budgeting (4 modules) |
| **Chatbot modules** | 37–39 total modules (3–6 per topic); each 5–10 minutes long; including introduction, quiz, comic, parenting tip, and home practice; personalized by caregiver’s and child’s gender and child’s age |
| **Delivery format** | Daily automated push of modules via chatbot on WeChat  Self-directed features in the chatbot:  – Choose topic sequence (after completing first topic of parent-child relationships)  – Set daily delivery time  – Choose text or video format for parenting tips |
| **Add-on human-led component** | Weekly or twice weekly message-based online group interactions on WeChat, facilitated by headteachers and social workers, on core tips and caregiver concerns (e.g., praise, device use, sexual safety) |
| **Additional features in the chatbot** | – On-demand troubleshooting  – Disability screening  – Safeguarding support  – Parenting tips library  – Playful parent–child activity library |

## Table 2. Outcome Measures

| **Outcome** | **Tool** | **Validation** | **Item**  **Number** | **Sample Item** | **Report and Scoring** |
| --- | --- | --- | --- | --- | --- |
| Early Learning and Stimulation | Multiple Indicator Cluster Surveys[6] | The Early Childhood Development Index 2030 (ECDI2030) constitutes the core developmental assessment component within the UNICEF Multiple Indicator Cluster Surveys (MICS) child development module. Although the present study did not administer the full ECDI2030 scale, the stimulation items used are embedded within this validated MICS framework. The ECDI2030 has been implemented and psychometrically evaluated in Chinese populations, demonstrating acceptable internal consistency (α ≈ 0.73)[7]. | 6 | How often in the past week did you read books to or look at picture books with (NAME)? | Caregiver report  A frequency between 0 and 7, or 8 or more times  Range: 0-48 |
| Caregiver-Perpetrated Violence | International Society for the Prevention of Child Abuse and Neglect Child Abuse Screening Tool-Parent version[8] | Psychometrically validated in the Chinese mainland among parents of preschool-aged children. Confirmatory factor analysis supports its multidimensional structure, with internal consistency coefficients ranging from 0.60 to 0.87 across subscales[9]. | 9 | How often did you discipline your child by slapping, spanking, or hitting with your hand? | Caregiver report  A frequency between 0 and 7, or 8 or more times  Range: 0-72, with higher score indicating worse outcome |
| Attitude towards Corporal Punishment | Multiple Indicator Cluster Surveys[6] |  | 1 | In order to bring up, raise up, or educate a child properly, the child needs to be physically punished. | Caregiver report  5-point Likert scale with options ranging from strongly disagree to strongly agree  Range: 1-5, with higher score indicating worse outcome |
| Child Behaviour | Strengths and Difficulties Questionnaire[10] | Validated in large-scale Chinese samples (n > 20,000), demonstrating acceptable reliability for the total difficulties score (α ≈ 0.69–0.73) and established construct validity across urban and rural populations[11]. | 25 | Often unhappy, depressed or tearful | Caregiver report  3-point Likert scale with options ranging from not true to certainly true  Total score range: 0-40, with higher score indicating worse outcome  Prosocial subscale score range: 0-10, with higher score indicating better outcome |
| Positive Parenting Practice | Alabama Parenting Questionnaire Positive Parenting and Involvement Subscales[12] | Validated in Chinese samples. Short-form versions demonstrate stable factor structures and acceptable reliability for Positive Parenting and Involvement subscales[13]. | 15 | You attend PTA meetings, parent/teacher conferences, or other meetings at your child’s school. | Caregiver report  5-point Likert scale with options ranging from never to always  Range: 0-75, with higher score indicating better outcome |
| Parental Mental Health | Depression, Anxiety, and Stress Scale-21 Depression and Anxiety Subscales[14] | Extensively validated in Chinese adult populations, demonstrating high internal consistency (α ≈ 0.95) and measurement invariance across gender[15]. | 14 | I found it hard to wind down. | Caregiver report  4-point Likert scale with options ranging from did not apply to apply most of the time  Range: 0-42, with higher score indicating more symptoms |
| Parenting Stress | Parental Stress Scale[16] | Demonstrated strong reliability (person reliability ≈ 0.86; Cronbach’s α ≈ 0.89) in both clinical and nonclinical Chinese parent samples[17]. | 18 | Caring for my child(ren) sometimes takes more time and energy than I have to give. | Caregiver report  5-point Likert scale with options ranging from strongly disagree to strongly agree  Range: 18-90, with higher score indicating more stress |
| Family Function | Family APGAR Scale[18] | Widely validated in Chinese populations, demonstrating excellent internal consistency (α ≈ 0.88–0.91) and strong convergent validity with measures of family communication and psychological well-being[19,20] | 5 | Are you satisfied that your family accepts and supports your wishes to take on new activities or directions? | Caregiver report  3-point Likert scale with options ranging from hardly to always  Range: 0-10, with higher score indicating better outcomes |

## Table 3a. Minimum Detectable Effect Size (Beta) under Linear Model for Early Learning and Stimulation

| ICC | Group size | | | |
| --- | --- | --- | --- | --- |
|  | 20 | 25 | 30 | 35 |
| 0.01 | 0.15 | 0.14 | 0.13 | 0.12 |
| 0.02 | 0.16 | 0.15 | 0.14 | 0.13 |
| 0.03 | 0.17 | 0.16 | 0.15 | 0.15 |
| 0.04 | 0.18 | 0.17 | 0.16 | 0.16 |
| 0.05 | 0.19 | 0.18 | 0.18 | 0.17 |

***Note.***Total cluster = 21; Significant level (α) = 0.05

## Table 3b. Minimum Detectable Effect Size (IRR) under Poisson Model for Caregiver-Perpetrated Violence

| ICC | Group size | | | |
| --- | --- | --- | --- | --- |
|  | 20 | 25 | 30 | 35 |
| 0.01 | 0.86 | 0.87 | 0.88 | 0.89 |
| 0.02 | 0.85 | 0.86 | 0.87 | 0.87 |
| 0.03 | 0.84 | 0.85 | 0.86 | 0.86 |
| 0.04 | 0.83 | 0.84 | 0.85 | 0.85 |
| 0.05 | 0.83 | 0.83 | 0.84 | 0.84 |

***Note.*** Total cluster = 21; Significant level (α) = 0.05

## Table 4. Demographic Characteristics and Baseline Measures of Participants by Group (T0)

|  | **Overall** | **Treatment** | **Control** |
| --- | --- | --- | --- |
|  | n=541 | n=272 | n=269 |
| **Caregiver Age (mean, SD)** | 36.6 (5.4) | 36.6 (5.2) | 36.7 (5.7) |
| **Caregiver Gender (N, %)** |  |  |  |
| Female | 404 (74.8) | 199 (73.2) | 205 (76.5) |
| Male | 136 (25.2) | 73 (26.8) | 63 (23.5) |
| **Relationship with the Child (N, %)** |  |  |  |
| Mother | 402 (74.3) | 199 (73.2) | 203 (75.5) |
| Father | 135 (25.0) | 71 (26.1) | 64 (23.8) |
| Grandparents | 3 (0.6) | 1 (0.4) | 2 (0.7) |
| Other | 1 (0.2) | 1 (0.4) | 0 (0.0) |
| **Ethnicity (N, %)** |  |  |  |
| Han | 538 (99.4) | 271 (99.6) | 267 (99.3) |
| Minorities | 3 (0.6) | 1 (0.4) | 2 (0.7) |
| **Hukou (Household Registration) (N, %)** |  |  |  |
| Rural | 177 (32.7) | 79 (29.0) | 98 (36.4) |
| Urban | 364 (67.3) | 193 (71.0) | 171 (63.6) |
| **Marital Status (N, %)** |  |  |  |
| Single | 6 (1.1) | 4 (1.5) | 2 (0.7) |
| Not single but unmarried | 2 (0.4) | 1 (0.4) | 1 (0.4) |
| Married | 526 (98.5) | 262 (98.1) | 264 (98.9) |
| **Employment (N, %)** |  |  |  |
| Full-time | 419 (80.4) | 216 (82.4) | 203 (78.4) |
| Part-time | 14 (2.7) | 8 (3.1) | 6 (2.3) |
| Unemployed | 35 (6.7) | 14 (5.3) | 21 (8.1) |
| Self-Employed | 45 (8.6) | 21 (8.0) | 24 (9.3) |
| Other | 8 (1.5) | 3 (1.1) | 5 (1.9) |
| **Caregiver Education (N, %)** |  |  |  |
| Primary school | 4 (0.7) | 2 (0.7) | 2 (0.7) |
| Middle school | 27 (5.0) | 12 (4.5) | 15 (5.6) |
| High school/Vocational school | 46 (8.6) | 19 (7.1) | 27 (10.1) |
| Junior college | 126 (23.6) | 64 (23.9) | 62 (23.2) |
| Undergraduate | 305 (57.0) | 155 (57.8) | 150 (56.2) |
| Postgraduate | 27 (5.0) | 16 (6.0) | 11 (4.1) |
| **Use of Digital Device (N, %)** |  |  |  |
| Rarely | 7 (1.3) | 2 (0.7) | 5 (1.9) |
| Sometimes | 184 (34.0) | 77 (28.3) | 107 (39.8) |
| Always | 350 (64.7) | 193 (71.0) | 157 (58.4) |
| **Child Age (mean, SD)** | 4.9 (0.9) | 4.8 (0.9) | 5.00 (0.9) |
| **Child Gender (N, %)** |  |  |  |
| Female | 234 (43.5) | 115 (42.6) | 119 (44.4) |
| Male | 302 (56.1) | 153 (56.7) | 149 (55.6) |
| **Number of Children in the Household (mean, SD)** | 1.9 (0.6) | 1.8 (0.5) | 1.9 (0.6) |
| **Number of People in the Household (mean, SD)** | 4.5 (1.1) | 4.5 (1.0) | 4.6 (1.1) |
| **Primary Caregiver (N, %)** |  |  |  |
| Mother | 413 (76.3) | 203 (74.6) | 210 (78.1) |
| Father | 215 (39.7) | 116 (42.6) | 99 (36.8) |
| Grandparents | 253 (46.8) | 137 (50.4) | 116 (43.1) |
| **Caregiver with Disability (N, %)** | 86 (15.9) | 41 (15.1) | 45 (16.7) |
| **Child with Disability (N, %)** | 128 (23.7) | 62 (22.8) | 66 (24.5) |

## Table 5. Participant Engagement in the Chatbot

| **Topic** | **N of Caregivers Who Completed the Topic** | **Percentage Of Caregivers Who Completed the Topic** | **N Of Caregivers Who Started the Topic** | **Completion Rate Based on Caregivers Who Started the Topic** |
| --- | --- | --- | --- | --- |
| Parent-Child Relationships | 212 | 77.94% | 260 | 81.54% |
| Child Development | 193 | 70.96% | 203 | 95.07% |
| Child Behavior Management | 185 | 68.01% | 194 | 95.36% |
| Proactive Parenting | 184 | 67.65% | 201 | 92.04% |
| Child Safety and Nutrition | 176 | 64.71% | 190 | 92.63% |
| Child Learning | 195 | 71.69% | 205 | 95.12% |
| Family Relationships | 179 | 65.81% | 190 | 94.21% |
| Parenting Budget | 173 | 63.60% | 179 | 96.65% |

## Fig 1. Distribution of Total Modules Completed Among Participants in the Intervention Group

**
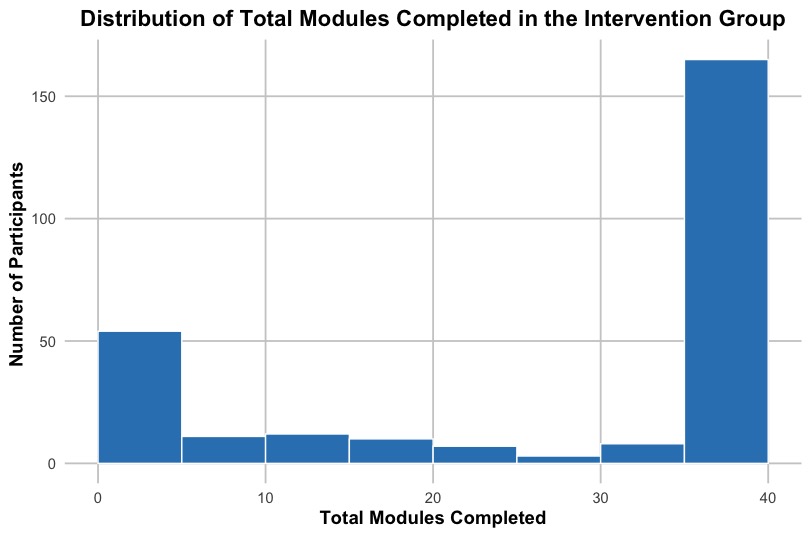
**

## Table 6. Means and Standard Deviations of Outcome Measures at Baseline, Immediate Post-Intervention

|  | **Treatment** | | **Control** | |
| --- | --- | --- | --- | --- |
|  | **Baseline** | **Post-Intervention** | **Baseline** | **Post-Intervention** |
|  | Mean (SD) | Mean (SD) | Mean (SD) | Mean (SD) |
| **Opportunities for Early Learning and Stimulation** | 21.86 (9.61) | 25.25 (8.83) | 21.74 (9.13) | 23.34 (8.26) |
| **Caregiver-Perpetrated violence: Total** | 14.04 (5.31) | 4.26 (4.52) | 14.44 (5.71) | 5.05 (5.63) |
| **Caregiver-Perpetrated violence: Physical** | 5.49 (2.38) | 1.28 (2.24) | 5.58 (2.66) | 1.84 (2.88) |
| **Caregiver-Perpetrated violence: Emotional** | 8.55 (3.52) | 2.98 (3.02) | 8.87 (3.96) | 3.21 (3.46) |
| **Attitude towards Corporal Punishment** | 2.60 (1.32) | 2.21 (1.24) | 2.76 (1.32) | 2.66 (1.31) |
| **Child Behaviour: Total** | 12.35 (4.21) | 9.04 (3.79) | 12.04 (3.85) | 9.28 (4.18) |
| **Child Behaviour:**  **Internalising behaviour** | 5.83 (2.09) | 3.16 (1.97) | 5.46 (2.23) | 3.34 (2.24) |
| **Child Behaviour: Externalising behaviour** | 6.51 (3.18) | 5.88 (2.77) | 6.58 (2.73) | 5.93 (2.94) |
| **Child Behaviour: Emotional problem** | 3.83 (1.43) | 1.55 (1.33) | 3.63 (1.40) | 1.74 (1.55) |
| **Child Behaviour: Conduct problem** | 2.33 (1.39) | 2.02 (1.24) | 2.18 (1.19) | 2.02 (1.32) |
| **Child Behaviour: Hyperactivity** | 4.18 (2.41) | 3.86 (2.14) | 4.40 (2.14) | 3.92 (2.24) |
| **Child Behaviour: Peer problem** | 2.00 (1.43) | 1.61 (1.20) | 1.83 (1.37) | 1.60 (1.29) |
| **Child Behaviour: Prosocial behaviour** | 7.26 (1.96) | 7.57 (1.89) | 7.36 (1.74) | 7.91 (1.76) |
| **Positive Parenting Practice: Total** | 56.51 (7.35) | 57.50 (7.33) | 57.21 (7.17) | 56.27 (7.15) |
| **Positive Parenting Practice:**  **Positive Parenting** | 23.96 (3.38) | 24.28 (3.19) | 24.06 (3.35) | 23.39 (3.03) |
| **Positive Parenting Practice:**  **Parental involvement** | 32.54 (4.94) | 33.22 (4.80) | 33.15 (4.70) | 32.88 (4.84) |
| **Parental Mental Health: Total** | 3.86 (5.64) | 3.30 (4.65) | 3.68 (5.22) | 4.25 (4.77) |
| **Parental Mental Health: Depression** | 2.01(3.26) | 1.83 (2.71) | 1.68 (2.96) | 1.62 (2.57) |
| **Parental Mental Health: Anxiety** | 1.84 (3.02) | 1.46 (2.52) | 2.00 (2.89) | 2.63 (2.78) |
| **Parenting Stress** | 38.36 (6.86) | 38.31 (7.02) | 38.66 (7.10) | 39.66 (6.66) |
| **Family Function** | 2.61 (2.41) | 2.98 (2.57) | 2.69 (2.56) | 2.42 (2.63) |

***Note.*** None of the above variables have missing values unless due to participant dropout. Attrition rates are shown in the Results section.

## Table 7. Distribution Check for Caregiver-Perpetrated Violence

|  | **AD test** | | **Dispersion test** | | | | **Distribution** |
| --- | --- | --- | --- | --- | --- | --- | --- |
| **Caregiver-Perpetrated Violence** | p-value raw | p-value log | ChiSq | Ratio | DF | p-value |  |
| Total | <0.001 | <0.001 | 1189.16 | 1.15 | 1038.00 | <0.001 | Negative Binomial |
| Physical | <0.001 | <0.001 | 1042.99 | 1.00 | 1038.00 | 0.451 | Poisson |
| Emotional | <0.001 | <0.001 | 877.34 | 0.85 | 1038.00 | 1.000 | Poisson |

## Table 8. Per-Protocol Sensitivity Analysis for Primary Outcomes

| **Outcome** | **Estimate** | **SE** | **95% CI** | **p-value** |
| --- | --- | --- | --- | --- |
| Early Learning and Stimulation | 2.46 | 0.88 | 0.74, 4.17 | 0.005 |
| Caregiver-Perpetrated Violence (Total) | 0.88 | 0.06 | 0.79, 0.99 | 0.026 |

## Table 9. Sensitivity Analyses Adjusting for Covariates: Intervention Effects on Primary and Secondary Outcomes

|  | Effect Size | 95% CI | P-Value |
| --- | --- | --- | --- |
| **Opportunities for Early Learning and Stimulation** | 1.73 | [0.15, 3.30] | 0.032 |
| **Caregiver-Perpetrated Violence: Total** | 0.88 | [0.80, 0.97] | 0.011 |
| **Caregiver-Perpetrated Violence: Physical** | 0.72 | [0.62, 0.85] | <0.001 |
| **Caregiver-Perpetrated Violence: Emotional** | 0.97 | [0.87, 1.09] | 0.646 |
| **Attitude towards Corporal Punishment** | -0.29 | [-0.53, -0.05] | 0.017 |
| **Child Behavior: Total** | -0.65 | [-1.31, 0.01] | 0.055 |
| **Child Behavior: Internalizing behavior** | -0.60 | [-1.00, -0.21] | 0.003 |
| **Child Behavior: Externalizing behavior** | -0.05 | [-0.50, 0.40] | 0.831 |
| **Child Behavior: Emotional problem** | -0.43 | [-0.69, -0.18] | 0.001 |
| **Child Behavior: Conduct problem** | -0.17 | [-0.42, 0.07] | 0.170 |
| **Child Behavior: Hyperactivity** | 0.13 | [-0.22, 0.48] | 0.467 |
| **Child Behavior: Peer problem** | -0.17 | [-0.44, 0.10] | 0.215 |
| **Child Behavior: Prosocial behavior** | -0.19 | [-0.51, 0.13] | 0.236 |
| **Parenting Practices: Total** | 2.25 | [0.89, 3.60] | 0.001 |
| **Parenting Practices: Positive parenting** | 1.11 | [0.44, 1.78] | 0.001 |
| **Parenting Practices: Parental involvement** | 1.14 | [0.27, 2.01] | 0.010 |
| **Parental Mental Health: Total** | -1.16 | [-2.03, -0.29] | 0.009 |
| **Parental Mental Health: Depression** | -0.18 | [-0.72, 0.37] | 0.527 |
| **Parental Mental Health: Anxiety** | -0.98 | [-1.45, -0.51] | <0.001 |
| **Parenting Stress** | -1.21 | [-2.48, 0.07] | 0.064 |
| **Family Functioning** | 0.61 | [0.14, 1.08] | 0.010 |

**Table 10a. Subgroup and Moderation Analyses by Caregiver Disability Status**

|  |  | **β/ IRR** | **SE** | **95% CI** | **P-Value** |
| --- | --- | --- | --- | --- | --- |
| Opportunities for Early Learning and Stimulation | 3-way interaction | -2·50 | 2·17 | [-6·77, 1·76] | 0·249 |
|  | caregiver without disabilities | 2·20 | 0·87 | [0·5, 3·91] | 0·011 |
|  | caregiver with disability | -0·37 | 1·84 | [-4·03, 3·28] | 0·839 |
| Caregiver-Perpetrated Violence: Total | 3-way interaction | 0·84 | 0·11 | [0·65, 1·07] | 0·157 |
|  | caregiver without disabilities | 0·91 | 0·00 | [0·91, 0·91] | <0·001 |
|  | caregiver with disability | 0·76 | 0·09 | [0·61, 0·95] | 0·016 |
| Caregiver-Perpetrated Violence: Physical | 3-way interaction | 0·71 | 0·14 | [0·47, 1·05] | 0·087 |
|  | caregiver without disabilities | 0·78 | 0·07 | [0·65, 0·93] | 0·007 |
|  | caregiver with disability | 0·55 | 0·10 | [0·39, 0·79] | 0·001 |
| Caregiver-Perpetrated Violence: Emotional | 3-way interaction | 0·96 | 0·15 | [0·71, 1·3] | 0·780 |
|  | caregiver without disabilities | 0·97 | 0·06 | [0·86, 1·1] | 0·662 |
|  | caregiver with disability | 0·93 | 0·13 | [0·7, 1·23] | 0·614 |
| Attitude towards Corporal Punishment | 3-way interaction | -0·54 | 0·33 | [-1·18, 0·1] | 0·096 |
|  | caregiver without disabilities | -0·21 | 0·13 | [-0·47, 0·04] | 0·101 |
|  | caregiver with disability | -0·76 | 0·28 | [-1·31, -0·2] | 0·009 |
| Child Behaviour: Total | 3-way interaction | 0·17 | 0·91 | [-1·62, 1·96] | 0·855 |
|  | caregiver without disabilities | -0·65 | 0·36 | [-1·36, 0·07] | 0·076 |
|  | caregiver with disability | -0·49 | 0·79 | [-2·05, 1·08] | 0·536 |
| Child Behaviour: Internalising behaviour | 3-way interaction | 0·17 | 0·55 | [-0·91, 1·24] | 0·758 |
|  | caregiver without disabilities | -0·61 | 0·21 | [-1·04, -0·19] | 0·004 |
|  | caregiver with disability | -0·44 | 0·52 | [-1·48, 0·6] | 0·404 |
| Child Behaviour: Externalising behaviour | 3-way interaction | 0·01 | 0·62 | [-1·2, 1·23] | 0·983 |
|  | caregiver without disabilities | -0·04 | 0·25 | [-0·52, 0·45] | 0·884 |
|  | caregiver with disability | -0·03 | 0·54 | [-1·1, 1·05] | 0·960 |
| Child Behaviour: Emotional problem | 3-way interaction | 0·13 | 0·35 | [-0·56, 0·82] | 0·715 |
|  | caregiver without disabilities | -0·44 | 0·14 | [-0·7, -0·17] | 0·002 |
|  | caregiver with disability | -0·30 | 0·36 | [-1·01, 0·41] | 0·405 |
| Child Behaviour: Conduct problem | 3-way interaction | 0·10 | 0·34 | [-0·56, 0·77] | 0·763 |
|  | caregiver without disabilities | -0·17 | 0·14 | [-0·44, 0·1] | 0·226 |
|  | caregiver with disabilities | -0·10 | 0·25 | [-0·6, 0·41] | 0·708 |
| Child Behaviour: Hyperactivity | 3-way interaction | -0·07 | 0·48 | [-1·01, 0·88] | 0·889 |
|  | caregiver without disabilities | 0·14 | 0·19 | [-0·23, 0·51] | 0·468 |
|  | caregiver with disabilities | 0·08 | 0·45 | [-0·82, 0·97] | 0·867 |
| Child Behaviour: Peer problem | 3-way interaction | 0·06 | 0·37 | [-0·67, 0·79] | 0·878 |
|  | caregiver without disabilities | -0·18 | 0·14 | [-0·46, 0·1] | 0·204 |
|  | caregiver with disabilities | -0·09 | 0·40 | [-0·88, 0·71] | 0·826 |
| Child Behaviour: Prosocial behaviour | 3-way interaction | 0·64 | 0·44 | [-0·22, 1·5] | 0·142 |
|  | caregiver without disabilities | -0·36 | 0·18 | [-0·71, -0·02] | 0·040 |
|  | caregiver with disabilities | 0·27 | 0·36 | [-0·44, 0·98] | 0·450 |
| Parenting Practices: Total | 3-way interaction | -0·50 | 1·92 | [-4·26, 3·26] | 0·795 |
|  | caregiver without disabilities | 2·10 | 0·77 | [0·58, 3·61] | 0·007 |
|  | caregiver with disabilities | 1·62 | 1·56 | [-1·48, 4·72] | 0·302 |
| Parenting Practices: Positive parenting | 3-way interaction | -0·20 | 0·93 | [-2·03, 1·64] | 0·832 |
|  | caregiver without disabilities | 1·02 | 0·37 | [0·29, 1·76] | 0·007 |
|  | caregiver with disabilities | 0·83 | 0·79 | [-0·75, 2·41] | 0·301 |
| Parenting Practices: Parental involvement | 3-way interaction | -0·30 | 1·23 | [-2·72, 2·12] | 0·806 |
|  | caregiver without disabilities | 1·08 | 0·49 | [0·11, 2·05] | 0·030 |
|  | caregiver with disabilities | 0·79 | 1·03 | [-1·26, 2·83] | 0·445 |
| Parental Mental Health: Total | 3-way interaction | -0·59 | 1·19 | [-2·93, 1·75] | 0·618 |
|  | caregiver without disabilities | -1·01 | 0·44 | [-1·87, -0·15] | 0·021 |
|  | caregiver with disabilities | -1·49 | 1·45 | [-4·39, 1·4] | 0·307 |
| Parental Mental Health: Depression | 3-way interaction | -0·30 | 0·75 | [-1·77, 1·16] | 0·685 |
|  | caregiver without disabilities | -0·09 | 0·27 | [-0·62, 0·45] | 0·751 |
|  | caregiver with disabilities | -0·35 | 0·93 | [-2·2, 1·5] | 0·706 |
| Parental Mental Health: Anxiety | 3-way interaction | -0·30 | 0·64 | [-1·56, 0·96] | 0·638 |
|  | caregiver without disabilities | -0·92 | 0·25 | [-1·4, -0·44] | <0·001 |
|  | caregiver with disabilities | -1·16 | 0·71 | [-2·57, 0·25] | 0·106 |
| Parenting Stress | 3-way interaction | 0·33 | 1·76 | [-3·13, 3·78] | 0·853 |
|  | caregiver without disabilities | -1·23 | 0·71 | [-2·62, 0·16] | 0·084 |
|  | caregiver with disabilities | -1·03 | 1·42 | [-3·85, 1·78] | 0·467 |
| Family Functioning | 3-way interaction | -1·28 | 0·64 | [-2·54, -0·02] | **0·047** |
|  | caregiver without disabilities | 0·83 | 0·26 | [0·33, 1·33] | 0·001 |
|  | caregiver with disabilities | -0·45 | 0·56 | [-1·57, 0·67] | 0·425 |

**Table 10b. Subgroup and Moderation Analyses by Child Disability Status**

|  |  | **β/ IRR** | **SE** | **95% CI** | **P-Value** |
| --- | --- | --- | --- | --- | --- |
| Opportunities for Early Learning and Stimulation | 3-way interaction | -1·97 | 1·87 | [-5·65, 1·71] | 0·294 |
|  | child without disabilities | 2·23 | 0·91 | [0·44, 4·01] | 0·014 |
|  | child with disabilities | 0·29 | 1·58 | [-2·84, 3·42] | 0·855 |
| Caregiver-Perpetrated Violence: Total | 3-way interaction | 0·90 | 0·10 | [0·73, 1·11] | 0·343 |
|  | child without disabilities | 0·91 | 0·05 | [0·81, 1·01] | 0·087 |
|  | child with disabilities | 0·85 | 0·10 | [0·68, 1·08] | 0·178 |
| Caregiver-Perpetrated Violence: Physical | 3-way interaction | 0·72 | 0·13 | [0·51, 1·03] | 0·070 |
|  | child without disabilities | 0·80 | 0·08 | [0·66, 0·96] | 0·020 |
|  | child with disabilities | 0·58 | 0·09 | [0·43, 0·77] | <0·001 |
| Caregiver-Perpetrated Violence: Emotional | 3-way interaction | 1·04 | 0·14 | [0·81, 1·35] | 0·747 |
|  | child without disabilities | 0·96 | 0·07 | [0·84, 1·1] | 0·542 |
|  | child with disabilities | 1·00 | 0·11 | [0·8, 1·24] | 0·998 |
| Attitude towards Corporal Punishment | 3-way interaction | 0·00 | 0·28 | [-0·55, 0·55] | 0·995 |
|  | child without disabilities | -0·29 | 0·13 | [-0·55, -0·03] | 0·030 |
|  | child with disabilities | -0·29 | 0·26 | [-0·8, 0·21] | 0·254 |
| Child Behaviour: Total | 3-way interaction | -0·37 | 0·79 | [-1·91, 1·17] | 0·639 |
|  | child without disabilities | -0·52 | 0·39 | [-1·28, 0·23] | 0·175 |
|  | child with disabilities | -0·96 | 0·63 | [-2·19, 0·28] | 0·129 |
| Child Behaviour: Internalising behaviour | 3-way interaction | -0·02 | 0·47 | [-0·94, 0·91] | 0·974 |
|  | child without disabilities | -0·58 | 0·22 | [-1·01, -0·14] | 0·009 |
|  | child with disabilities | -0·60 | 0·44 | [-1·47, 0·27] | 0·175 |
| Child Behaviour: Externalising behaviour | 3-way interaction | -0·38 | 0·53 | [-1·43, 0·67] | 0·480 |
|  | child without disabilities | 0·06 | 0·26 | [-0·45, 0·57] | 0·828 |
|  | child with disabilities | -0·34 | 0·44 | [-1·21, 0·53] | 0·441 |
| Child Behaviour: Emotional problem | 3-way interaction | 0·46 | 0·30 | [-0·13, 1·06] | 0·129 |
|  | child without disabilities | -0·51 | 0·15 | [-0·8, -0·23] | <0·001 |
|  | child with disabilities | -0·06 | 0·26 | [-0·58, 0·47] | 0·832 |
| Child Behaviour: Conduct problem | 3-way interaction | 0·16 | 0·29 | [-0·41, 0·73] | 0·582 |
|  | child without disabilities | -0·19 | 0·14 | [-0·47, 0·08] | 0·173 |
|  | child with disabilities | -0·06 | 0·25 | [-0·55, 0·44] | 0·816 |
| Child Behaviour: Hyperactivity | 3-way interaction | -0·50 | 0·42 | [-1·32, 0·31] | 0·225 |
|  | child without disabilities | 0·25 | 0·20 | [-0·14, 0·64] | 0·206 |
|  | child with disabilities | -0·24 | 0·37 | [-0·97, 0·48] | 0·510 |
| Child Behaviour: Peer problem | 3-way interaction | -0·48 | 0·32 | [-1·11, 0·15] | 0·136 |
|  | child without disabilities | -0·06 | 0·15 | [-0·35, 0·22] | 0·663 |
|  | child with disabilities | -0·55 | 0·32 | [-1·18, 0·09] | 0·090 |
| Child Behaviour: Prosocial behaviour | 3-way interaction | 0·44 | 0·38 | [-0·3, 1·18] | 0·248 |
|  | child without disabilities | -0·37 | 0·18 | [-0·72, -0·01] | 0·044 |
|  | child with disabilities | 0·07 | 0·33 | [-0·58, 0·72] | 0·833 |
| Parenting Practices: Total | 3-way interaction | 1·19 | 1·65 | [-2·05, 4·43] | 0·471 |
|  | child without disabilities | 1·72 | 0·79 | [0·17, 3·26] | 0·029 |
|  | child with disabilities | 2·93 | 1·48 | [0, 5·86] | 0·050 |
| Parenting Practices: Positive parenting | 3-way interaction | 0·63 | 0·81 | [-0·95, 2·21] | 0·433 |
|  | child without disabilities | 0·85 | 0·39 | [0·08, 1·61] | 0·030 |
|  | child with disabilities | 1·50 | 0·70 | [0·11, 2·88] | 0·034 |
| Parenting Practices: Parental involvement | 3-way interaction | 0·55 | 1·06 | [-1·53, 2·63] | 0·604 |
|  | child without disabilities | 0·88 | 0·50 | [-0·11, 1·86] | 0·081 |
|  | child with disabilities | 1·43 | 0·97 | [-0·49, 3·36] | 0·143 |
| Parental Mental Health: Total | 3-way interaction | 0·94 | 1·04 | [-1·11, 2·99] | 0·369 |
|  | child without disabilities | -1·24 | 0·48 | [-2·18, -0·31] | 0·009 |
|  | child with disabilities | -0·29 | 1·05 | [-2·37, 1·79] | 0·780 |
| Parental Mental Health: Depression | 3-way interaction | 0·95 | 0·65 | [-0·32, 2·22] | 0·143 |
|  | child without disabilities | -0·31 | 0·29 | [-0·87, 0·25] | 0·280 |
|  | child with disabilities | 0·66 | 0·70 | [-0·72, 2·04] | 0·344 |
| Parental Mental Health: Anxiety | 3-way interaction | 0·03 | 0·56 | [-1·07, 1·13] | 0·957 |
|  | child without disabilities | -0·94 | 0·26 | [-1·46, -0·43] | <0·001 |
|  | child with disabilities | -0·90 | 0·55 | [-1·98, 0·18] | 0·103 |
| Parenting Stress | 3-way interaction | 0·60 | 1·52 | [-2·38, 3·57] | 0·694 |
|  | child without disabilities | -1·27 | 0·71 | [-2·66, 0·12] | 0·073 |
|  | child with disabilities | -0·74 | 1·45 | [-3·61, 2·13] | 0·609 |
| Family Functioning | 3-way interaction | -0·65 | 0·56 | [-1·74, 0·44] | 0·242 |
|  | child without disabilities | 0·79 | 0·26 | [0·28, 1·29] | 0·002 |
|  | child with disabilities | 0·17 | 0·54 | [-0·9, 1·25] | 0·752 |

## Table 11. Changes in Outcome Variables from Baseline to Post-Intervention and 12-Month Follow-Up in the Intervention Group

| **Outcome** | **Time Point** | **β/ IRR** | **SE** | **95% CI** | **P-Value** |
| --- | --- | --- | --- | --- | --- |
| Early Learning and Stimulation | T1 | 3.36 | 0.53 | [2.32, 4.40] | <0.001 |
|  | T2 | 6.70 | 0.56 | [5.60, 7.80] | <0.001 |
|  | T3 | 2.37 | 0.62 | [1.14, 3.59] | <0.001 |
| Caregiver-Perpetrated Violence: Total | T1 | 0.27 | 0.05 | [0.24, 0.29] | <0.001 |
|  | T2 | 0.38 | 0.05 | [0.34, 0.42] | <0.001 |
|  | T3 | 0.34 | 0.06 | [0.30, 0.38] | <0.001 |
| Caregiver-Perpetrated Violence: Physical | T1 | 0.23 | 0.06 | [0.21, 0.26] | <0.001 |
|  | T2 | 0.26 | 0.06 | [0.23, 0.30] | <0.001 |
|  | T3 | 0.27 | 0.07 | [0.23, 0.31] | <0.001 |
| Caregiver-Perpetrated Violence: Emotional | T1 | 0.35 | 0.04 | [0.32, 0.38] | <0.001 |
|  | T2 | 0.55 | 0.04 | [0.51, 0.60] | <0.001 |
|  | T3 | 0.50 | 0.05 | [0.46, 0.54] | <0.001 |
| Attitude towards Corporal Punishment | T1 | -0.38 | 0.08 | [-0.54, -0.23] | <0.001 |
|  | T2 | 0.39 | 0.08 | [0.22, 0.55] | <0.001 |
|  | T3 | 0.52 | 0.09 | [0.33, 0.70] | <0.001 |
| Child Behaviour: Total | T1 | -3.32 | 0.23 | [-3.78, -2.87] | <0.001 |
|  | T2 | -3.64 | 0.24 | [-4.12, -3.16] | <0.001 |
|  | T3 | -3.94 | 0.29 | [-4.51, -3.38] | <0.001 |
| Child Behaviour: Internalising behaviour | T1 | -2.67 | 0.14 | [-2.95, -2.40] | <0.001 |
|  | T2 | -2.60 | 0.15 | [-2.89, -2.31] | <0.001 |
|  | T3 | -2.89 | 0.16 | [-3.22, -2.57] | <0.001 |
| Child Behaviour: Externalising behaviour | T1 | -0.64 | 0.17 | [-0.98, -0.30] | <0.001 |
|  | T2 | -1.02 | 0.18 | [-1.38, -0.66] | <0.001 |
|  | T3 | -1.04 | 0.20 | [-1.44, -0.64] | <0.001 |
| Child Behaviour: Emotional problem | T1 | -2.28 | 0.09 | [-2.46, -2.10] | <0.001 |
|  | T2 | -2.20 | 0.10 | [-2.39, -2.01] | <0.001 |
|  | T3 | -2.39 | 0.11 | [-2.6, -2.18] | <0.001 |
| Child Behaviour: Conduct problem | T1 | -0.31 | 0.09 | [-0.49, -0.13] | 0.001 |
|  | T2 | -0.34 | 0.09 | [-0.53, -0.16] | <0.001 |
|  | T3 | -0.50 | 0.11 | [-0.71, -0.29] | <0.001 |
| Child Behaviour: Hyperactivity | T1 | -0.33 | 0.13 | [-0.59, -0.07] | 0.012 |
|  | T2 | -0.67 | 0.14 | [-0.94, -0.40] | <0.001 |
|  | T3 | -0.54 | 0.15 | [-0.84, -0.24] | <0.001 |
| Child Behaviour: Peer problem | T1 | -0.39 | 0.10 | [-0.58, -0.20] | <0.001 |
|  | T2 | -0.40 | 0.10 | [-0.59, -0.20] | <0.001 |
|  | T3 | -0.51 | 0.11 | [-0.73, -0.29] | <0.001 |
| Child Behaviour: Prosocial behaviour | T1 | 0.28 | 0.11 | [0.05, 0.50] | 0.016 |
|  | T2 | 0.59 | 0.12 | [0.35, 0.83] | <0.001 |
|  | T3 | 0.69 | 0.14 | [0.42, 0.95] | <0.001 |
| Parenting Practices: Total | T1 | 0.94 | 0.48 | [0, 1.88] | 0.051 |
|  | T2 | 1.66 | 0.51 | [0.67, 2.66] | 0.001 |
|  | T3 | 1.21 | 0.56 | [0.10, 2.31] | 0.033 |
| Parenting Practices: Positive parenting | T1 | 0.29 | 0.23 | [-0.16, 0.74] | 0.209 |
|  | T2 | 0.69 | 0.24 | [0.22, 1.16] | 0.004 |
|  | T3 | 0.18 | 0.27 | [-0.35, 0.70] | 0.506 |
| Parenting Practices: Parental involvement | T1 | 0.65 | 0.32 | [0.03, 1.28] | 0.039 |
|  | T2 | 0.97 | 0.33 | [0.32, 1.63] | 0.004 |
|  | T3 | 1.04 | 0.37 | [0.31, 1.77] | 0.005 |
| Parental Mental Health: Total | T1 | -0.45 | 0.29 | [-1.02, 0.13] | 0.130 |
|  | T2 | -2.31 | 0.31 | [-2.92, -1.70] | <0.001 |
|  | T3 | -0.48 | 0.35 | [-1.16, 0.20] | 0.163 |
| Parental Mental Health: Depression | T1 | -0.16 | 0.18 | [-0.52, 0.20] | 0.388 |
|  | T2 | -1.24 | 0.19 | [-1.62, -0.86] | <0.001 |
|  | T3 | -0.22 | 0.22 | [-0.65, 0.20] | 0.302 |
| Parental Mental Health: Anxiety | T1 | -0.29 | 0.15 | [-0.59, 0.01] | 0.059 |
|  | T2 | -1.08 | 0.16 | [-1.4, -0.76] | <0.001 |
|  | T3 | -0.26 | 0.18 | [-0.62, 0.10] | 0.151 |
| Parenting Stress | T1 | 0.01 | 0.51 | [-0.98, 1.01] | 0.976 |
|  | T2 | -2.56 | 0.53 | [-3.61, -1.51] | <0.001 |
|  | T3 | 4.10 | 0.59 | [2.93, 5.26] | <0.001 |
| Family Functioning | T1 | 0.37 | 0.15 | [0.07, 0.66] | 0.017 |
|  | T2 | -0.27 | 0.16 | [-0.59, 0.04] | 0.088 |
|  | T3 | 0.09 | 0.18 | [-0.26, 0.44] | 0.606 |

***Note.*** T1 = post-intervention assessment; T2 = 6-month follow-up; T3 = 12-month follow-up. IRR = incidence rate ratio; SE = standard error; CI = confidence interval.
Caregiver-Perpetrated Violence: Total was analysed using a negative binomial multilevel regression model due to overdispersion. Caregiver-Perpetrated Violence: Physical and Emotional were analysed using Poisson multilevel regression models. All other outcomes were analysed using linear mixed-effects models.

## Table 12. Baseline Characteristics by 12-Month Follow-Up Status in the Intervention Group

| **Variable** | **Dropout (n=116)** | **Completer (n=156)** | **p-value** |
| --- | --- | --- | --- |
| Early Learning and Stimulation | 20.61 (9.74) | 22.81 (9.41) | 0.063 |
| Caregiver-Perpetrated Violence (Total) | 14.05 (5.35) | 14.04 (5.31) | 0.984 |
| Child Age | 5.99 (1.01) | 5.38 (0.92) | <0.001 |
| Caregiver Age | 37.34 (4.86) | 36.05 (5.35) | 0.041 |
| Caregiver Gender |  |  | 0.004 |
| – Female | 42 (36.2%) | 31 (19.9%) |  |
| – Male | 74 (63.8%) | 125 (80.1%) |  |
| Employment Status |  |  | 0.021 |
| – Full-time | 100 (86.2%) | 116 (74.4%) |  |
| – Part-time | 1 (0.9%) | 7 (4.5%) |  |
| – Unemployed | 2 (1.7%) | 12 (7.7%) |  |
| – Self-employed | 7 (6.0%) | 14 (9.0%) |  |

## Table 13. Baseline Predictors of Compliance in the Intervention Group

| **Variable** | **Mean (Non-Complier)** | **Mean (Complier)** | **OR** | **CI Low** | **CI High** | **P-Value** |
| --- | --- | --- | --- | --- | --- | --- |
| Child Gender | 0.32 | 0.49 | 2.09 | 1.24 | 3.57 | 0.006 |
| Caregiver Gender (ref: boy) | 1.66 | 1.77 | 1.71 | 0.99 | 2.97 | 0.054 |
| Child Age | 5.72 | 5.60 | 0.89 | 0.69 | 1.14 | 0.356 |
| Caregiver Age | 37.16 | 36.32 | 0.97 | 0.92 | 1.02 | 0.207 |
| Opportunities for Early Learning and Stimulation | 20.89 | 22.41 | 1.02 | 0.99 | 1.04 | 0.211 |
| Caregiver-Perpetrated Violence: Total | 14.13 | 14.04 | 1.00 | 0.95 | 1.05 | 0.890 |
| Caregiver-Perpetrated Violence: Physical | 5.64 | 5.43 | 0.96 | 0.87 | 1.07 | 0.485 |
| Caregiver-Perpetrated Violence: Emotional | 8.49 | 8.61 | 1.01 | 0.94 | 1.09 | 0.792 |
| Attitude towards Corporal Punishment | 2.65 | 2.58 | 0.96 | 0.80 | 1.16 | 0.695 |
| Child Behavior: Total | 12.76 | 12.20 | 0.97 | 0.91 | 1.03 | 0.285 |
| Child Behavior: Internalizing behavior | 5.86 | 5.84 | 1.00 | 0.89 | 1.13 | 0.964 |
| Child Behavior: Externalizing behavior | 6.91 | 6.35 | 0.95 | 0.87 | 1.02 | 0.167 |
| Child Behavior: Emotional problem | 3.88 | 3.82 | 0.97 | 0.82 | 1.16 | 0.760 |
| Child Behavior: Conduct problem | 2.41 | 2.31 | 0.95 | 0.79 | 1.13 | 0.545 |
| Child Behavior: Hyperactivity | 4.49 | 4.05 | 0.93 | 0.83 | 1.03 | 0.142 |
| Child Behavior: Peer problem | 1.98 | 2.02 | 1.02 | 0.86 | 1.22 | 0.808 |
| Child Behavior: Prosocial behavior | 6.89 | 7.46 | 1.16 | 1.02 | 1.32 | 0.023 |
| Parenting Practices: Total | 54.99 | 57.21 | 1.04 | 1.01 | 1.08 | 0.017 |
| Parenting Practices: Positive parenting | 23.47 | 24.18 | 1.07 | 0.99 | 1.15 | 0.100 |
| Parenting Practices: Parental involvement | 31.52 | 33.03 | 1.07 | 1.01 | 1.12 | 0.016 |
| Parental Mental Health: Total | 4.54 | 3.48 | 0.97 | 0.93 | 1.01 | 0.147 |
| Parental Mental Health: Depression | 2.41 | 1.79 | 0.95 | 0.87 | 1.02 | 0.140 |
| Parental Mental Health: Anxiety | 2.12 | 1.69 | 0.96 | 0.88 | 1.04 | 0.262 |
| Parenting Stress | 40.18 | 37.34 | 0.94 | 0.90 | 0.98 | 0.001 |
| Family Functioning | 3.03 | 2.40 | 0.90 | 0.81 | 0.99 | 0.040 |

## Table 14. Estimated Complier Average Causal Effects on Primary and Secondary Outcome Variables

We estimated the complier average causal effect (CACE) using a two-stage instrumental variable (IV) approach, treating random assignment as an instrument for compliance.

In the first stage, compliance was defined as completing at least 30 intervention modules. Compliance status was regressed on randomization to generate predicted compliance probabilities for each participant.

In the second stage, each outcome was modelled as a function of the predicted compliance indicator, using mixed-effects regression models with random intercepts at both the cluster and participant levels to account for clustering and repeated measures. Continuous outcomes were analyzed using linear mixed-effects models, and count outcomes were analyzed using Poisson or negative binomial mixed-effects models, as appropriate.

This two-stage IV framework provides an unbiased estimate of the causal effect of the intervention among compliers, thereby addressing non-adherence while preserving the benefits of randomization.

|  | **Β/ IRR** | **SE** | **95% CI** | **P-Value** |
| --- | --- | --- | --- | --- |
| Opportunities for Early Learning and Stimulation | 1.33 | 1.63 | [-2.11, 4.77] | 0.427 |
| Caregiver-Perpetrated Violence: Total | 0.93 | 0.08 | [0.86,1.09] | 0.393 |
| Caregiver-Perpetrated Violence: Physical | 0.92 | 0.08 | [0.86, 1.08] | 0.264 |
| Caregiver-Perpetrated Violence: Emotional | 0.98 | 0.09 | [0.86,1.10] | 0.795 |
| Attitude towards Corporal Punishment | -0.45 | 0.16 | [-0.78, -0.11] | 0.011 |
| Child Behavior: Total | 0.35 | 0.86 | [-1.46, 2.16] | 0.689 |
| Child Behavior: Internalizing behavior | 0.24 | 0.43 | [-0.67, 1.15] | 0.587 |
| Child Behavior: Externalizing behavior | 0.06 | 0.53 | [-1.06, 1.17] | 0.916 |
| Child Behavior: Emotional problem | 0.07 | 0.23 | [-0.38, 0.53] | 0.756 |
| Child Behavior: Conduct problem | 0.17 | 0.23 | [-0.31, 0.65] | 0.462 |
| Child Behavior: Hyperactivity | -0.13 | 0.36 | [-0.89, 0.62] | 0.714 |
| Child Behavior: Peer problem | 0.17 | 0.26 | [-0.37, 0.7] | 0.52 |
| Child Behavior: Prosocial behavior | -0.43 | 0.31 | [-1.08, 0.21] | 0.178 |
| Parenting Practices: Total | 0.27 | 1.07 | [-1.96, 2.51] | 0.803 |
| Parenting Practices: Positive parenting | 0.60 | 0.56 | [-0.56, 1.76] | 0.291 |
| Parenting Practices: Parental involvement | -0.30 | 0.66 | [-1.67, 1.07] | 0.653 |
| Parental Mental Health: Total | -0.49 | 0.69 | [-1.93, 0.96] | 0.49 |
| Parental Mental Health: Depression | 0.44 | 0.39 | [-0.39, 1.27] | 0.275 |
| Parental Mental Health: Anxiety | -0.92 | 0.42 | [-1.8, -0.05] | 0.040 |
| Parenting Stress | -1.41 | 1.08 | [-3.66, 0.84] | 0.205 |
| Family Functioning | 0.39 | 0.43 | [-0.52, 1.3] | 0.374 |

## Table 15. Cluster-Level Compliance Rates in the Intervention Group

| **Cluster** | **N Total** | **N of Complier** | **Complier Rate** |
| --- | --- | --- | --- |
| 15 | 28 | 28 | 100.00% |
| 9 | 22 | 21 | 92.30% |
| 2 | 26 | 24 | 91.30% |
| 12 | 34 | 27 | 77.10% |
| 10 | 33 | 24 | 72.70% |
| 4 | 23 | 14 | 60.90% |
| 7 | 15 | 6 | 40.00% |
| 5 | 23 | 9 | 39.10% |
| 17 | 31 | 10 | 32.30% |
| 20 | 35 | 10 | 28.60% |

## Methods. Pragmatic Trial Orientation

The study was designed to reflect pragmatic trial principles. Eligibility criteria were broad, with minimal exclusion conditions. The intervention was embedded within routine preschool services and delivered primarily through existing school structures. Headteachers, as regular preschool staff, played an active role in facilitating group discussions and supporting caregiver engagement. Social workers were introduced to support implementation fidelity and to guide headteachers in becoming familiar with the intervention procedures, rather than to provide specialized therapeutic delivery.

Participation procedures were integrated into existing communication platforms, flexibility in engagement was permitted, and primary outcomes reflected real-world parenting behaviors. Analyses followed the intention-to-treat principle. Collectively, these features position the trial toward the pragmatic end of the explanatory–pragmatic continuum, consistent with domains outlined in the PRECIS-2 framework [21].

## References:

[1] Shenderovich Y, Lachman JM, Ward CL, Wessels I, Gardner F, Tomlinson M, et al. The science of scale for violence prevention: A new agenda for family strengthening in low-and middle-income countries. Front Public Health 2021;9:581440.

[2] Ward CL, Wessels IM, Lachman JM, Hutchings J, Cluver L, Kassanjee R, et al. Parenting for Lifelong Health for Young Children: A randomized controlled trial of a parenting program in South Africa to prevent harsh parenting and child conduct problems. J Child Psychol Psychiatry 2020;61:503–12. https://doi.org/10.1111/jcpp.13129.

[3] Lachman JamieM, Alampay LP, Jocson R, Alinea MCD, Madrid B, Ward CL, et al. Effectiveness of a parenting programme to reduce violence in a cash transfer system in the Philippines: RCT with follow-up. Lancet Reg Health West Pac 2021;17:100279.

[4] Moore G, Campbell M, Copeland L, Craig P, Movsisyan A, Hoddinott P, et al. Adapting interventions to new contexts—the ADAPT guidance. BMJ 2021;374. https://doi.org/10.1136/BMJ.N1679.

[5] Barrera M, Berkel C, Castro FG. Directions for the Advancement of Culturally Adapted Preventive Interventions: Local Adaptations, Engagement, and Sustainability. Prev Sci 2017;18:640–8. https://doi.org/10.1007/s11121-016-0705-9.

[6] Khan S, Hancioglu A. Multiple Indicator Cluster Surveys: Delivering Robust Data on Children and Women across the Globe. Stud Fam Plann 2019;50:279–86. https://doi.org/10.1111/sifp.12103.

[7] Zou S, Zou X, Zhang R, Xue K, Xiao AY, Zhou M, et al. Maternal depression and early childhood development among children aged 24–59 months: the mediating effect of responsive caregiving. Ann Gen Psychiatry 2024;23:30. https://doi.org/10.1186/s12991-024-00515-z.

[8] Meinck F, Boyes ME, Cluver L, Ward CL, Schmidt P, DeStone S, et al. Adaptation and psychometric properties of the ISPCAN Child Abuse Screening Tool for use in trials (ICAST-Trial) among South African adolescents and their primary caregivers. Child Abuse Negl 2018;82:45–58. https://doi.org/10.1016/j.chiabu.2018.05.022.

[9] Chen C, Wang X, Qin J, Huang Z. Psychometric testing of the Chinese version of ISPCAN Child Abuse Screening Tools Parent’s version (ICAST-P). Child Youth Serv Rev 2020;109.

[10] Du Y, Kou J, Coghill D. The validity, reliability and normative scores of the parent, teacher and self report versions of the Strengths and Difficulties Questionnaire in China. Child Adolesc Psychiatry Ment Health 2008;2:8. https://doi.org/10.1186/1753-2000-2-8.

[11] Gao X, Shi W, Zhai Y, He L, Shi X. Results of the parent-rated Strengths and Difficulties Questionnaire in 22,108 primary school students from 8 provinces of China. Shanghai Arch Psychiatry 2013;25:364–74. https://doi.org/10.3969/j.issn.1002-0829.2013.06.005.

[12] Frick PJ. The Alabama parenting questionnaire. Univ Ala 1991.

[13] Hsieh M. Reliability and validity of the Chinese version of the Short-form Alabama Parenting Questionnaire. Arch Guid Couns 2020.

[14] Chan RCK, Xu T, Huang J, Wang Y, Zhao Q, Shum DHK, et al. Extending the utility of the Depression Anxiety Stress scale by examining its psychometric properties in Chinese settings. Psychiatry Res 2012;200:879–83. https://doi.org/10.1016/j.psychres.2012.06.041.

[15] Jiang L, Yan Y, Jin Z-S, Hu M-L, Wang L, Song Y, et al. The Depression Anxiety Stress Scale-21 in Chinese Hospital Workers: Reliability, Latent Structure, and Measurement Invariance Across Genders. Front Psychol 2020;11:247. https://doi.org/10.3389/fpsyg.2020.00247.

[16] Berry JO, Jones WH. The Parental Stress Scale: Initial psychometric evidence. J Soc Pers Relatsh 1995:463–72. https://doi.org/10.1177/0265407595123009.

[17] Leung C, Tsang SKM. The Chinese Parental Stress Scale: Psychometric Evidence Using Rasch Modeling on Clinical and Nonclinical Samples. J Pers Assess 2010;92:26–34. https://doi.org/10.1080/00223890903379209.

[18] Smilkstein G, Ashworth C, Montano D. Validity and reliability of the family APGAR as a test of family function. J Fam Pract 1982;15:303–11.

[19] Wang D, Zhu F, Xi S, Niu L, Tebes JK, Xiao S, et al. Psychometric Properties of the Multidimensional Scale of Perceived Social Support (MSPSS) Among Family Caregivers of People with Schizophrenia in China. Psychol Res Behav Manag 2021;14:1201–9. https://doi.org/10.2147/PRBM.S320126.

[20] Nan H, Ni MY, Lee PH, Tam WWS, Lam TH, Leung GM, et al. Psychometric evaluation of the Chinese version of the subjective happiness scale: Evidence from the Hong Kong family cohort. Int J Behav Med 2014;21:646–52. https://doi.org/10.1007/s12529-014-9389-3.

[21] Loudon K, Treweek S, Sullivan F, Donnan P, Thorpe KE, Zwarenstein M. The PRECIS-2 tool: designing trials that are fit for purpose 2015. https://doi.org/10.1136/bmj.h2147.
