# Supplementary material for: Effectiveness of a universal digital–human parenting intervention in promoting early childhood development and protection: A pragmatic cluster randomized controlled trial
Source: PLOS Digit Health. 2026 May 7;5(5):e0001357. doi: 10.1371/journal.pdig.0001357 (PMC13152119; doi:10.1371/journal.pdig.0001357)
Supplement: S1 File — (DOCX) [file pdig.0001357.s003.docx]

**Effectiveness of a universal digital–human parenting intervention in promoting early childhood development and protection: A pragmatic cluster randomized controlled trial**

**Introduction**

Evidence shows that approximately one billion children aged 2 to 17 globally have experienced violence in the past year [1]. Research in China also indicates that 20% to 27% children aged under 18 have experienced physical and emotional violent discipline [2]. Violence against children (VAC) is a global public health issue, which can result in both immediate and enduring negative consequences on child cognitive development and mental and physical health, as well as substantial societal costs.

Early childhood is a pivotal period characterized by rapid brain development. It plays a vital role in fostering holistic child development and unlocking children’s full potential [3]. Despite being a critical window of opportunity, this developmental stage is also characterized by vulnerability, as children are particularly susceptible to the adverse effects of childhood adversities, including a lack of responsive caregiving and exposure to violence.

Parenting programs are designed to promote positive parenting behaviours and reducing violence against children perpetrated by caregivers. Global evidence has shown the effectiveness of these programs in improving a range of factors associated with violent discipline, such as child behaviour, parent-child relationships, and parental mental health [4–7]. Previous research has also demonstrated their potential in promoting some aspects of early childhood development (ECD), including social skills, motor skills, communication abilities, and cognitive development [8–12]. In recent years, there has been a growing trend toward digital parenting interventions, aimed at expanding program reach and scalability. Some evidence from developed countries suggests favorable outcomes across various child and adult indicators [13,14]. However, there remains a lack of research involving large samples to assess the effectiveness of such interventions in developing countries, where the need may be more pronounced.

This study aims to evaluate the implementation and effectiveness of a digital parenting program, called Keyushiguang, on altering the incidence of and factors associated with child development and protection. Keyushiguang has been culturally adapted from Parenting for Lifelong Health ParentText Young Children for delivery via WeChat in China. A hybrid approach, which combines chatbot-led digital delivery and human-led online interactions, will be used to deliver the intervention for Chinese families of preschool children.

**Objectives**

Objective 1: To evaluate the effect of the hybrid parenting intervention "Keyushiguang" on reducing emotional and physical violent discipline and promoting early learning and stimulation, at immediate post-intervention, in comparison to a waitlist control group.

Objective 2: To evaluate the effect of the hybrid parenting intervention "Keyushiguang" on reducing child behavioral problems, increasing positive parenting, improving parental mental health, reducing parenting stress, changing parental attitude towards violent discipline, and improving family function at immediate post-intervention, in comparison to a waitlist control group.

Objective 3: To investigate the feasibility (including implementation fidelity, participant engagement, and acceptability of the hybrid parenting intervention.

Objective 4: To understand the sustainability of program effects at 6-month and 1-year follow-ups.

**Study Design**

A randomized controlled trial with two parallel groups will be conducted to examine the effectiveness of Keyushiguang. The randomization procedure will be conducted using a random sequence generator in Excel. To ensure allocation concealment, the process will be carried out by an independent researcher who is not familiar with the trial, with minimal involvement of the research. 21 classes, with approximately 30 children per class, in a preschool will be recruited and randomized at cluster (class) level into the treatment and waitlist control groups.

A mixed-methods approach will be used with a nested design, in which the qualitative approach will be embedded within the quantitative method to better understand the quantitative data obtained. The study will be conducted within the context of routine services. Data will be collected at baseline (T0), immediate post-intervention (T1), 6-month post-intervention (T2), and 1-year post-intervention (T3). The study is expected to start in March 2024 and will last until June 2025.

The trial protocol was prospectively registered on the Chinese Clinical Trial Registry (ChiCTR2400081911).

**Participants**

Participating caregivers/parents will be those who: 1) are adults aged 18 years or older; 2) are primary caregivers of a child aged between 3 to 6 or enrolled in a preschool; 3) have access to a smartphone; and 4) consent to participate in the research and the implementation of the intervention. Program facilitators will be those who: 1) are adults aged 18 years or older; 2) are trained on the content and delivery of Keyushiguang; 3) have access to a smartphone; and 4) consent to participate in the research and implementation of the intervention.

Families will be recruited from a preschool in Xinyu, Jiangxi Province, China. Facilitators will be recruited from the preschool's head teachers and from the master of social work program at Beijing Normal University.

**Timeline**

| **Activity** | **Timeline** |
| --- | --- |
| Participant recruitment | March 2024 |
| Eligibility screening and randomization | March 2024 |
| Consent procedure and baseline assessment | March 2024 |
| Implementation | March to May 2024 |
| Post-intervention assessment | May 2024 |

**Intervention**

Keyushiguang is adapted from the PLH ParentText Young Children program and is grounded in social learning theory and attachment theory. The program consists of eight primary goals, including parent-child relationships, child development, child behavior, child learning, proactive parenting, child safety, family relationships, and parenting budget, each comprising three to six modules. Each module is dedicated to a specific positive parenting principle or technique. Keyushiguang delivers parenting knowledge through an interactive chatbot, with a frequency of one module per day, lasting between five to ten minutes each. Additionally, a home activity is assigned at the conclusion of each module, with a follow-up home activity check-in sent the following day. The program lasts for approximately 2.5 months.

Participants will be organized into small groups. Each group will be overseen by two trained program facilitators, consisting of a preschool teacher and a qualified social worker. These facilitators will guide regular interactions, reinforce the acquired knowledge, and encourage active engagement among caregivers.

**Ethics**

Oral informed consent will be obtained from all participants. Confidentiality will be maintained unless the participant indicates that he or she will conduct self-injurious behaviors, or there are any concerns about possible child abuse events, for which appropriate action will be taken in adherence to the Child Protection Protocol and in terms of informing the relevant authority. Ethics approval was granted by Beijing Normal University (SSDPP-HSC-2024003).

**Outcomes and Measures**

Outcome evaluation-primary outcome:

Caregiver-perpetrated physical and emotional violence will be measured using parent report of a short version of the ICAST-TP. Respondents will be asked to report the frequency of emotional and physical violent disciplinary practices over the past two weeks using a 9-point frequency count scale.

Child early stimulation and responsive caregiving will be measured using six items from the MICS early childhood development section.

Outcome evaluation-secondary outcomes:

Child behaviors will be measured using parent report of the Strengths and Difficulties Questionnaire.

Parental mental health will be assessed using the self-report Depression and Anxiety subscales of the Depression Anxiety, and Stress Scale-21.

Parenting stress will be measured using the self-report Parental Stress Scale.

Positive parenting will be assessed using the Alabama Parenting Questionnaire Positive Parenting and Involvement subscales.

Attitude towards corporal punishment will be measured using one item from the MICS survey, asking participants whether they believe that in order to bring up, raise up, or educate a child properly, the child needs to be physically punished.

Family function will be assessed using the Chinese version of the Family APGAR scale.

Feasibility:

Implementation fidelity will be assessed by facilitators filling out a weekly fidelity survey.

Participant engagement with the chatbot will be monitored by a technical team, who will gather all user interactions with the chatbot. Examples are the enrolment and completion rates of the program, goals, modules, home activities, and check-in questions.

Acceptability will be assessed using a participant satisfaction questionnaire and through qualitative interviews with caregivers and facilitators.

**Data collection**

All data collectors will be trained. The four data collection time points include baseline, immediate post-intervention, six months after the end of delivery, and one year after the end of delivery. Data collectors will administer the questionnaires with parents through a video-conferencing platform. Data will be collected using encrypted online questionnaires.

Qualitative individual interviews will be conducted with approximately 20 parents, with the final number depending on when data saturation is reached. Interviewees will be purposively sampled according to the engagement rates and satisfaction levels. Each interview will last for a maximum of 90 minutes. Interviews with parents will be undertaken online after the completion of the program. Focus groups will be conducted with around ten facilitators online at post-intervention, with each lasting for a maximum of two hours. A semi-structured interview schedule will be developed to guide the interviews and focus groups. Open-ended questions will be used to stimulate reflection and discussion.

Due to the nature of parent training and the use of a waitlist control group, it will not be possible to blind participants or facilitators. However, research staff who collect the data will be blinded for the baseline and immediate post-intervention assessment. Data collectors will not be involved in the delivery process and will be trained on the topic of risk of bias. Parents will also be asked to keep their allocation condition private from outcome data collectors. Process evaluation data will be collected by a separate group of research staff. Blinding will be protected and monitored, while the violation of blinding will be detected and incorporated into the interpretation of results.

Electronic data will be stored in organizational OneDrive that meets university security requirements. Only the core research team will have access to the data. All data will also be backed up in an encrypted hard drive that is only available to the core research team. All data will be pseudonymized. An ID number will be assigned to each participant after they give consent. The linkage between the ID number and participant personal details will be stored with other electronic data. Data will be managed and processed using Excel and R Studio.

**Data Analysis**

Quantitative data analyses will be conducted using R Studio. Data analysis will follow the principles of intent-to-treat, which involves assessing all participants as originally allocated, regardless of program dropouts or the dose of treatment they received.

A table showing the demographic characteristics of participants will be compiled (M±SD for continuous variables and percentages for categorical variables). Baseline differences in demographic characteristics and outcome measures will be explored using two independent samples t-tests and chi-square tests (significant level p<0.05).

The primary analysis will comprise a between-group comparison for the treatment and the waitlist control groups immediately after the intervention (T1) to investigate the treatment effects. Distribution tests will be run to determine the appropriate regression model. Exploratory statistical analyses will be conducted to understand the differences in treatment effects across different demographic characteristics. Mediator and moderator analyses might be conducted to investigate potential pathways of treatment effects. Means and standard deviations will be calculated for the feasibility measures.

Interview data will be transcribed and coded using ATLAS.ti 8. A thematic analysis will be carried out using a contextualist framework, which is a mixture of essentialist and constructionist perspectives and combines the traditionally data-driven approach with a theory driven approach. A coding framework will be developed according to the topics covered by the interviews and focus group discussions. Transcripts will be read and re-read to generate initial codes, which will be organized into the priori framework. Initial codes will then be grouped into potential themes regarding participant involvement, program acceptability, delivery, and sustainability. The codes and themes will be reviewed by another research member, together with whom, the themes will be refined, defined, and reported as key themes. Pseudo anonymized data will be extracted to present and discuss the themes.

**Dissemination**

A brief report of study results in plain language will be shared with all participants. The study will result in several publications in peer-reviewed journals and presentations in regional and international conferences. The findings will be used to inform program modification.

**Monitoring**

Any potential harm or adverse events identified by the staff will be reported to the principal investigator, who will investigate the event and report to ethics committees before deciding whether to continue the study.

**Data Sharing**

Anonymized data will be shared upon request.

**Conflict of Interest**

There is no conflict of interest.

**References**

1. Hillis S, Mercy J, Amobi A, Kress H. Global prevalence of past-year violence against children: A systematic review and minimum estimates. *Pediatrics*. 2016;137(3):e20154079. doi:10.1542/peds.2015-4079
2. Fang X, Fry DA, Ji K, et al. The burden of child maltreatment in China: a systematic review. *Bull World Health Organ*. 2015;93(3):176-185C. doi:10.2471/BLT.14.140970
3. Richter LM, Daelmans B, Lombardi J, et al. Investing in the foundation of sustainable development: pathways to scale up for early childhood development. *Lancet*. 2017;389(10064):103-118. doi:10.1016/S0140-6736(16)31698-1
4. Flujas-Contreras JM, García-Palacios A, Gómez I. Technology-based parenting interventions for children’s physical and psychological health: a systematic review and meta-analysis. *Psychol Med*. 2019;49(11):1787-1798. doi:10.1017/S0033291719000692
5. Gardner F, Montgomery P, Knerr W. Transporting Evidence-Based Parenting Programs for Child Problem Behavior (Age 3–10) Between Countries: Systematic Review and Meta-Analysis. *J Clin Child Adolesc Psychol*. 2016;45(6):749-762. doi:10.1080/15374416.2015.1015134
6. Leijten P, Melendez-Torres GJ, Knerr W, Gardner F. Transported versus Homegrown Parenting Interventions for Reducing Disruptive Child Behavior: A Multilevel Meta-Regression Study. *J Am Acad Child Adolesc Psychiatry*. 2016;55(8):659-667.e3. doi:10.1016/j.jaac.2016.05.003
7. WHO. *WHO guidelines on parenting interventions to prevent maltreatment and enhance parent–child relationships with children aged 0–17 years*. Published 2023. Accessed September 4, 2025.
8. Benzies KM, Magill-Evans JE, Hayden KA, Ballantyne M. Key components of early intervention programs for preterm infants and their parents: a systematic review and meta-analysis. *BMC Pregnancy Childbirth*. 2013;13(suppl 1):S10. doi:10.1186/1471-2393-13-S1-S10
9. Britto PR, Ponguta L, Reyes C, Karnati R. *A Systematic Review of Parenting Programmes for Young Children in Low-and Middle-Income Countries*. United Nations Children’s Fund; 2015.
10. Geeraert L, Van Den Noortgate W, Grietens H, Onghena P. The effects of early prevention programs for families with young children at risk for physical child abuse and neglect: A meta-analysis. *Child Maltreat*. 2004;9(3):277-291. doi:10.1177/1077559504264265
11. Jeong J, Franchett EE, Ramos de Oliveira CV, Rehmani K, Yousafzai AK. Parenting interventions to promote early child development in the first three years of life: A global systematic review and meta-analysis. *PLoS Med*. 2021;18(5):e1003602. doi:10.1371/JOURNAL.PMED.1003602
12. Fang Z, Liu X, Zhang C, Lachman JM, Qiao D. Parenting Interventions That Promote Child Protection and Development for Preschool-Age Children with Developmental Disabilities: A Global Systematic Review and Meta-Analysis. *Trauma Violence Abuse*. 2024;25(3):2128-2142. doi:10.1177/15248380231207965
13. Corralejo SM, Domenech Rodríguez MM. Technology in Parenting Programs: A Systematic Review of Existing Interventions. *J Child Fam Stud*. 2018;27(9):2717-2731. doi:10.1007/S10826-018-1117-1
14. Thongseiratch T, Leijten P, Melendez-Torres GJ. Online parent programs for children’s behavioral problems: a meta-analytic review. *Eur Child Adolesc Psychiatry*. 2020;29(11):1555-1568. doi:10.1007/S00787-020-01472-0
