# Supplementary material for: Effectiveness of a universal digital–human parenting intervention in promoting early childhood development and protection: A pragmatic cluster randomized controlled trial
Source: PLOS Digit Health. 2026 May 7;5(5):e0001357. doi: 10.1371/journal.pdig.0001357 (PMC13152119; doi:10.1371/journal.pdig.0001357)
Supplement: S2 Text — (DOCX) [file pdig.0001357.s004.docx]

**Effectiveness of a universal digital–human parenting intervention in promoting early childhood development and protection: A pragmatic cluster randomized controlled trial**

**Statistical Analysis Plan**

**1. General Principles**

All primary analyses will be conducted following the Intention-to-Treat (ITT) principle. All participants will be analyzed in the groups to which they were originally assigned, regardless of the level of intervention completion or dosage received.

Unless otherwise specified, a p-value of < 0.05 will be considered statistically significant for all analyses.

**2. Sample Size and Power Calculation**

As this is a pragmatic trial, the sample size is primarily determined by real-world logistical constraints. While initial estimates suggested access to approximately 79 participants per group, the decision to further embed the intervention into the routine parenting support offered by the preschool allowed us to access all families within the setting.

To maximize the available sample, based on recruitment capacity, power calculations will assume 21 clusters, each comprising 20–35 participants (four discrete values), and intra-cluster correlation coefficients (ICCs) ranging from 0.01 to 0.05 (five values).

Prior to implementation, power calculations will be conducted to estimate the minimum detectable effect size (MDES) for the primary outcome, for opportunities for early learning and stimulation and caregiver-perpetrated violence, under fixed sample size constraints. MDES estimates will be based on a multilevel Poisson/negative binomial and a linear regression framework.

**3. Intervention Effect Estimation**

The primary evaluation of intervention effects will be conducted at the post-intervention timepoint (T1).

**3.1 Continuous Outcomes**

Continuous outcomes will be analyzed using multilevel linear regression models.

- **Model Specification:**
  - **Random Effects:** Participant ID nested within cluster.
  - **Fixed Effects:** Group (intervention vs. control), time (T0, T1, T2, T3), and the group × time interaction term. The intervention effect will be determined by the interaction term.

**3.2 Frequency-Based Outcomes**

Frequency-based outcomes, such as caregiver-perpetrated violence, will be analyzed using either Poisson or negative binomial regression models. The choice of model will be determined by the presence of overdispersion in the data.

- **Model Specification:** The model structure will be identical to that described in section 3.1, including random and fixed effects.

**3.3 Sensitivity Analyses**

To assess the robustness of the findings, sensitivity analyses will be conducted by adjusting the primary models for a set of pre-specified covariates.

- **Covariates:** Child age, child gender, caregiver age, and caregiver gender.

**4. Subgroup and Moderation Analyses**

To examine whether intervention effects differ by demographic status, moderation analyses will be conducted.

- **Model Specification:** A three-way interaction term (group × time × demographic status) will be added to the original models.

**5. Follow-up Analysis in the Intervention Group**

Due to the waitlist control design, the control group began receiving the intervention after the post-intervention (T1) assessment. Therefore, between-group comparisons at the 6-month (T2) and 12-month (T3) follow-ups are not possible.

- **Method:** Analyses will be restricted to the intervention group data.
- **Generalized linear mixed-effects models** will be applied, using baseline (T0) as the reference point.
- **Model Specification:**
  - **Random Effect:** Participant ID nested within cluster.
  - **Fixed Effect:** Time (T1, T2, T3) will be included as a fixed effect to assess changes in outcomes from baseline.

**6. Causal Effect Among Compliers**

The Complier Average Causal Effect (CACE) will be estimated using a two-stage instrumental variable (IV) approach, with random assignment serving as the instrument for compliance.

- **Stage 1:** Compliance status (defined as completing at least 30 chatbot modules) will be regressed on the randomization variable to generate predicted compliance probabilities for each participant.
- **Stage 2:** Each outcome will be modelled as a function of the predicted compliance indicator from Stage 1. This stage will use mixed-effects regression models with random intercepts at both the cluster and participant levels to account for data clustering and repeated measures.
  - Continuous outcomes will be analyzed using linear mixed-effects models.
  - Count outcomes will be analyzed using Poisson or negative binomial mixed-effects models, as appropriate.

This two-stage IV framework is designed to provide an unbiased estimate of the causal effect of the intervention among compliers, addressing non-adherence while preserving the benefits of randomization.

**7. Relationship to Trial Protocol and Statement of Deviation**

This Statistical Analysis Plan (SAP) serves as a detailed and specific elaboration of the data analysis principles outlined in the original trial protocol. The protocol provided a high-level summary of the analytical intentions, such as adhering to the intent-to-treat principle and conducting between-group comparisons.

This SAP operationalizes those intentions by pre-specifying the exact statistical models, covariates, and analytical methods to be used. Key specifications in this SAP that expand upon the protocol include the use of mixed-effects models to appropriately account for the clustered data structure and the precise definition of moderation and CACE analyses.

There are no deviations from the core aims or analytical principles described in the original trial protocol.
