# Supplementary material for: Effectiveness of a universal digital–human parenting intervention in promoting early childhood development and protection: A pragmatic cluster randomized controlled trial
Source: PLOS Digit Health. 2026 May 7;5(5):e0001357. doi: 10.1371/journal.pdig.0001357 (PMC13152119; doi:10.1371/journal.pdig.0001357)
Supplement: Attachment — Submitted filename: Response to Reviewers.docx [file pdig.0001357.s006.docx]

Dear Editor and Reviewers,

Thank you for the opportunity to revise our manuscript. We are grateful for the thoughtful and detailed feedback provided. The comments were highly constructive and have substantially strengthened the manuscript.

We have carefully revised the paper to address all concerns raised. A detailed, point-by-point response follows, with all revisions clearly marked in the manuscript.

We appreciate the time and expertise invested in reviewing our work and hope that the revised version meets the journal’s expectations.

Very Best Wishes,

The Authors

**Additional Editor Comments (if provided):**

Thank you for the opportunity to review this manuscript. The study, a pragmatic cluster RCT in a Chinese city, explores the Effectiveness of a universal digital–human parenting intervention in promoting early childhood development and protection. The study has several strengths, including the application of an adequate methodology to assess a novel intervention under real-life conditions. According to the authors, this is the first such study within the Chinese preschool system. Below are opportunities to improve the manuscript by addressing major and minor concerns:

Major:

1. Lines 121-129: Nicely presented knowledge gaps addressed by this study

Authors: Thank you!

2. Methods sections should precede the Results

Authors: Thank you for this helpful suggestion. In the originally submitted version, we placed the Materials and Methods section after the Results in accordance with the journal’s submission guidelines (https://journals.plos.org/digitalhealth/s/submission-guidelines#loc-headings). However, following your recommendation, we reviewed several recently published articles in PLOS Digital Health and noted that many present the Methods section before the Results.

We have therefore reorganized the manuscript accordingly. To facilitate clear review of other textual revisions, this relocation was done without using track changes. No substantive modifications were made to the content of the Methods section during this reordering.

3. Given the choice to offer the intervention to the control group, did the study team consider a formal cross-over design? Please justify your final design choice.

Authors: Thank you for your question. During the planning stage, we did consider alternative designs, including a formal cluster cross-over model. However, several conceptual and statistical considerations led us to adopt a parallel cluster randomized controlled trial with a waitlist control.

The primary reason relates to the expected persistence of intervention effects. The program targets parenting knowledge, attitudes toward corporal punishment, and parenting practices, which are not transient exposures and cannot be “washed out” between periods. A cross-over design relies on the assumption that intervention effects do not carry over into subsequent periods, or that a washout phase can restore baseline conditions. In the context of a behavioral parenting intervention, such an assumption would not be feasible. Once caregivers acquire and begin applying new parenting strategies, these changes are expected to persist, thereby violating the independence of periods required for valid cross-over inference.

We have clarified this design rationale in the revised Methods and Material section:

“A cross-over design was not adopted due to the expected persistence of intervention effects, which would violate the assumption of no carryover between periods.” (Line 153-154)

4. Parent input in the study seems central given the scope of the intervention. Please describe your family engagement strategy for this study.

Authors: Thank you for highlighting the central role of family engagement. Our study incorporated a structured, multi-level family engagement strategy embedded within the preschool system. We have now elaborated the strategy in the revised Methods section:

“Given that caregiver participation was central to intervention delivery, engagement was approached as a structured implementation component. The strategy operated at multiple levels.

At the institutional level, the program was introduced and endorsed by headteachers, who maintained established and trusted relationships with families. Positioning the intervention within routine preschool parenting support services helped legitimize participation and reduce stigma.

At the relational level, weekly or twice-weekly online group discussions facilitated by headteachers and social workers provided human interaction, opportunities for guided reflection, and social accountability. Embedding the intervention within WeChat class groups integrated participation into routine school–family communication channels, which lowered access barriers and normalizing engagement.

At the motivational level, a child-centered token incentive mechanism was implemented. Children received small developmentally appropriate rewards linked to caregiver engagement with chatbot modules, which indirectly reinforced sustained participation. Regular reminders and encouragement were delivered through established communication structures.

Together, these strategies aimed to enhance initial uptake, support adherence, and minimize attrition under real-world implementation conditions.” (Line 206-227)

5. Further describe if clusters (classrooms) were recruited before randomization, and who consented for their participation.

Authors: Thank you. We have clarified these procedures in the Methods section:

“The preschool institution agreed to participate prior to trial initiation, and all 21 eligible classes were recruited before randomization. Institutional approval for cluster participation was provided by the preschool administration. Randomization was conducted after baseline caregiver consent was completed.” (Line 173-177)

6. Recommend using a tool such as the PRECIS 2 framework that scores the study across multiple design domains, and helps assess where the trial falls on the pragmatic/explanatory continuum.

Authors: Thank you for your suggestion. We agree that the PRECIS-2 framework provides a structured approach to characterizing the pragmatic versus explanatory orientation of a trial. While we did not conduct a formal PRECIS-2 scoring exercise at the design stage, the trial incorporated several pragmatic features.

Eligibility criteria were broad, the intervention was embedded within routine preschool services, and headteachers (existing school staff) were actively involved in program delivery. Social workers supported implementation primarily to facilitate initial delivery and guide headteachers in familiarizing themselves with the intervention procedures, rather than functioning as specialized staff.

To clarify the trial’s positioning along the pragmatic-explanatory continuum, we have now added a brief description in the Appendices outlining how the study aligns with key PRECIS-2 domains.

“Appendix 15 Pragmatic Trial Orientation

The study was designed to reflect pragmatic trial principles. Eligibility criteria were broad, with minimal exclusion conditions. The intervention was embedded within routine preschool services and delivered primarily through existing school structures. Headteachers, as regular preschool staff, played an active role in facilitating group discussions and supporting caregiver engagement. Social workers were introduced to support implementation fidelity and to guide headteachers in becoming familiar with the intervention procedures, rather than to provide specialized therapeutic delivery.

Participation procedures were integrated into existing communication platforms, flexibility in engagement was permitted, and primary outcomes reflected real-world parenting behaviors. Analyses followed the intention-to-treat principle. Collectively, these features position the trial toward the pragmatic end of the explanatory–pragmatic continuum, consistent with domains outlined in the PRECIS-2 framework [13].”

7. In the methods, describe adherence to CONSORT and relevant extension to this study design: CONSORT extensions for cluster RCTs and for pragmatic trials. Follow their recommended nomenclature and organization study components

Authors: Thank you for this important recommendation. We have revised the manuscript to explicitly state adherence to the CONSORT 2025 statement and the relevant CONSORT extensions for cluster randomized trials and pragmatic trials.

“This study is reported in accordance with the CONSORT 2025 statement and the CONSORT extensions for cluster randomized trials and pragmatic trials [26–28].. Reporting has been aligned with the recommended nomenclature and organization of study components.” (Line 332-334)

The Methods section has been reviewed to ensure alignment with the recommended terminology and reporting structure, such as clarification of the rationale for the cluster design, cluster-level eligibility, reporting of cluster numbers in analysis, and consideration of pragmatic trial features.

8. Lines 448-452: has the PLH program been previously validated? Also provide more detail on the adaptation to Keyushiguang and any validation work in the manuscript, particularly with respect to linguistic and cultural adaptation.

Authors: Thank you. The Parenting for Lifelong Health (PLH) program is grounded in social learning theory and attachment theory and has been evaluated in more than 20 randomized controlled trials across diverse low- and middle-income settings. We clarified this theoretical and empirical foundation in Appendix Table 1.

Keyushiguang represents a culturally adapted version of PLH ParentText Young Children for implementation in China. We have expanded the description of the adaptation process in Appendix Table 1.

“Jul 2023 – Feb 2024, guided by ADAPT framework:[4,5]

-	Surface adaptations: linguistic translation, localization of visuals, examples, and metaphors.

-	Deep adaptations: content modifications to reflect local culture, e.g., filial piety, budgeting, communication norms, dietary practices.

-	The process involved literature and policy review, stakeholder consultations with caregivers, preschool teachers, and social workers, expert review panels, iterative content revision, and small-scale feasibility testing prior to trial implementation.

-	Care was taken to preserve the core behavior change principles of the original PLH intervention while enhancing contextual relevance and acceptability.”

9. What information, if any, did you collect on eligible participants who refused to participate?

Authors: Thank you for your question. No individual-level data were collected from caregivers who declined participation, in order to respect privacy and avoid collecting data without consent. As recruitment was conducted through routine preschool communication channels, only the number of eligible caregivers invited and the number who provided consent were recorded.

We have clarified this in the Methods section:

*“No demographic or baseline data were collected from caregivers who declined participation, in order to* respect *privacy and ethical considerations.” (Line 167-168)*

10. How did headteacher decide on whom to invite for participation? Could this have introduced a selection bias?

Authors: Thank you for raising this concern. Headteachers did not selectively recruit participants. All eligible caregivers of children enrolled in the participating classes were invited to take part in the study through routine preschool communication channels. No additional screening or discretionary selection was applied at the classroom level.

Participation was voluntary, and caregivers provided informed consent prior to enrollment. As with most pragmatic trials embedded in routine service settings, self-selection at the individual level cannot be entirely ruled out. However, the enrolment rate was 81.35%, suggesting broad uptake among eligible families.

We have clarified the recruitment procedures in the revised Methods section:

*“Headteachers invited all eligible caregivers within their respective classes through routine school communication channels.” (Line 165-166)*

11. Line 462 / Outcomes section: provide additional information in your manuscript on the validity and relevance of the selected scales, particularly with regards to family-centered outcomes. Focus on validation in the study setting. Also highlight who was conducting the assessments (self-report?; probability of under-reporting among abusive caregivers)\

Authors: Thank you for your suggestion. We have expanded Appendix Table 2: Outcome Measures to provide additional details regarding validation in the Chinese context.

We have also clarified in the Methods section:

*“All instruments used in this study have been translated and psychometrically evaluated in Chinese populations, with evidence supporting their reliability and construct validity in family and child development research contexts.” (Line 258-260)*

We have further acknowledged the potential bias in the Discussion section:

*“As outcomes relied on caregiver self-report, under-reporting due to social desirability bias cannot be excluded, particularly for sensitive behaviors such as violent discipline.” (Line 582-584)*

Minor:

12. Line 94: define what is meant by “stunting”

Authors: Thank you for this clarification request. We have now defined stunting:

*“…due to stunting (defined as height-for-age more than two standard deviations below the WHO Child Growth Standards median) or poverty.” (Line 94-95)*

13. Lines 95-6: provide references for these claims

Authors: Thank you for pointing this out. We have now added two key references to support these statements:

- Britto PR, Lye SJ, Proulx K, Yousafzai AK, Matthews SG, Vaivada T, et al. Nurturing care: promoting early childhood development. The Lancet 2017;389:91–102. https://doi.org/10.1016/S0140-6736(16)31390-3.
- Sayre RK, Devercelli AE, Neuman MJ, Wodon Q. Investing in early childhood development : Review of the world bank’s recent experience. World Bank 2015.

14. Line 460: “A 6-month follow-up (T2) was conducted with the intervention” ; clarify what is meant by intervention. Were all groups now receiving the intervention?

Authors: Thank you for highlighting this ambiguity. We have clarified the wording in the revised manuscript. The 6-month (T2) and 12-month (T3) follow-up assessments were conducted only with participants originally allocated to the intervention group.

Following the post-intervention assessment (T1), the waitlist control group began receiving the intervention and was therefore no longer available as a comparison group for longer-term follow-up.

*“A 6-month follow-up (T2) was conducted in December 2024 among the original intervention group, as the waitlist control group began receiving the intervention after T1. A 12-month follow-up (T3) with the same group was completed in June–July 2025.” (Line 242-245)*

15. Could you also provide per protocol effect estimates as a sensitivity analysis of you primary outcome estimates?

Authors: Thank you for your suggestion. We have now conducted a per-protocol sensitivity analysis for the primary outcomes. The per-protocol results were consistent with the ITT findings. A description of the per-protocol analysis has been added to the Methods section, and the corresponding results are reported in the Results section (page 15) and Appendix Table 13.

*“In addition to the pre-specified ITT and CACE analyses, a per-protocol sensitivity analysis was conducted. Participants in the intervention group who completed fewer than 30 chatbot modules were excluded, consistent with the compliance threshold defined in the Statistical Analysis Plan. Multilevel models identical to those used in the primary ITT analyses were applied to the restricted dataset.” (Line 327-331)*

*“Per-protocol analyses yielded results consistent with the ITT findings. Among participants who completed at least 30 modules, the intervention effect on Early Learning and Stimulation was β = 2.46 (95% CI 0.74–4.17, p = 0.005), and the incidence rate ratio for Caregiver-Perpetrated Violence (Total) was 0.88 (95% CI 0.79–0.99, p = 0.026) (see Appendix Table 13).” (Line 407-411)*

16. How was cluster-level rather than participant-level noncompliance under ITT? Was this an issue in the study?

Authors: Thank you for your question. There was no cluster-level noncompliance or crossover in this trial. All clusters randomized to the intervention arm received the intervention as allocated, and no control clusters were exposed to intervention components prior to the post-intervention assessment (T1). Noncompliance occurred only at the participant level, reflected in variation in module completion within intervention clusters.

The intention-to-treat analyses retained all participants in their originally randomized clusters, thereby preserving the cluster-randomized structure of the trial.

To improve clarity, we have added this information to the Methods section.

*“There was no cluster-level crossover or noncompliance during the trial. All clusters randomized to the intervention arm received the intervention as allocated. Variation in adherence occurred only at the participant level.” (Line 187-190)*

17. Are effects evaluated only at T1 or across all follow ups? Your model includes all time points, but the text implies T1 is the focus. Please clarify.

Authors: Thank you for highlighting this important point. The primary between-group effectiveness evaluation was conducted at post-intervention (T1), as the waitlist control group began receiving the intervention immediately after T1 and was therefore no longer available as a comparison group for later time points.

Although the statistical models were specified to include time as a factor, between-group comparisons were interpreted at T1 only. Follow-up analyses at T2 and T3 were conducted within the original intervention group to assess sustainability of effects relative to baseline.

We have clarified this distinction in both the Methods sections.

*“All models specified participant ID nested within cluster (class) as random effects to account for between-class variability, including potential differences in headteachers and facilitation dynamics, and included fixed effects for group (intervention vs control), time (T0, T1, T2, T3; primary inference for between-group effectiveness was restricted to T1), and their interaction (group × time).” (Line 298-303)*

18. How were missing data handled?

Authors: Thank you for your question. We have now clarified the handling of missing data in the Statistical Analysis section.

*“Caregivers were free to decline participation before starting the survey. During survey administration, data collectors asked caregivers to answer all items and to flag any questions they found unclear or awkward; when this occurred caregivers discussed their interpretation with the data collector (or referred the issue to the research team) and then provided the most appropriate response based on their understanding. As a result, item-level missing data were negligible by design. Any missing outcome data resulted from participant attrition at follow-up assessments and were handled using multilevel mixed-effects models estimated via maximum likelihood under standard missing-at-random assumptions. No multiple imputation was performed.” (Line 270-278)*

19. Sample size: specify what intracluster correlation you assumed in your calculations

Authors: Thank you for highlighting this. The assumed intra-cluster correlation coefficients (ICCs) used in the power calculations were previously reported in Supplementary Appendix 3.

For clarity and ease of reference, we have now moved this information into the Methods section:

*“Power calculations were conducted prior to implementation to estimate the minimum detectable effect size (MDES) for the primary outcomes under the fixed design constraints of the trial. Calculations assumed 21 clusters with 20–35 participants per cluster and intra-cluster correlation coefficients (ICCs) ranging from 0.01 to 0.05, reflecting values commonly reported in preschool-based and parenting intervention cluster trials. A two-sided significance level of α = 0.05 was applied.*

*Under these assumptions, the MDES for Early Learning and Stimulation (linear multilevel model) ranged from β = 0.12 to 0.19 depending on cluster size and ICC values. For Caregiver-Perpetrated Violence (Poisson/negative binomial multilevel models), the MDES ranged from IRR = 0.83 to 0.89. Full details of the power calculations are provided in S1 Appendix Table 3a and Table 3b.” (Line 282-292)*

20. Lines 497-99: But why wasn’t the control group followed for at least 6-months post the intiation of the intervention, even if the follow-up period would have been shorter than the original intervention group?

Authors: Thank you for your question. The primary objective of the trial was to evaluate effectiveness through a between-group comparison at T1 and to assess sustainability of effects among the original intervention group over a 12-month period.

Following T1, the waitlist control group began receiving the intervention as part of routine preschool programming. Extending follow-up assessments for the control group after their delayed intervention would have required a staggered longitudinal design across different cohorts, which was not feasible within the pragmatic constraints of the preschool calendar and available implementation resources.

In addition, class composition changes over the academic year (e.g., grade progression and transitions) would have complicated cluster integrity and long-term follow-up comparability.

We have clarified this design rationale in the Discussion-Limitations paragraph:

*“In addition, although the waitlist control group began receiving the intervention after T1, extended follow-up of this group was not conducted due to pragmatic constraints related to the preschool academic calendar and cohort transitions. As such, sustainability effects were evaluated within the original intervention group only.” (Line 595-598)*

21. Figure 3: clarify what the y axes represent

Authors: Thank you for your suggestion. Figure 3 has been revised to clearly specify that the y-axes represent the observed mean raw scores at each timepoint. The left panel now indicates the mean score for Early Learning and Stimulation, and the right panel indicates the mean score for Caregiver-Perpetrated Violence.

22. Line 273: “better family functioning (OR=0.90, 95%CI [0.81, 0.99], p=0.040) were associated with lower odds of compliance”. This seems counter-intuitive. How did you explain this finding?

Authors: Thank you for raising this point. We have verified the coding and direction of the family functioning variable, and the reported association is correct.

One possible explanation is that caregivers reporting higher baseline family functioning may have perceived less need for additional parenting support, which resulted in lower motivation to complete the full set of modules. This pattern is consistent with universal prevention programs, where families experiencing fewer challenges may engage less intensively due to lower perceived benefit.

We have now added this interpretation to the Discussion section to clarify the potential mechanism underlying this finding”

*“Interestingly, better baseline family functioning was associated with lower odds of compliance. One possible explanation is that caregivers in more well-functioning families perceived less need for additional parenting support, leading to lower engagement intensity. This pattern may reflect differential perceived need within universal intervention settings.” (Line 573-576)*

23. Note limitations between knowledge-based (what participants know/think is best) and behavior-based outcomes (objectively determined measure of what was actually done) due to the study design and outcome assessment.

Authors: Thank you for your suggestion. We have now clarified this limitation in the Discussion section:

*“Moreover, although the study included both attitudinal measures (e.g., endorsement of corporal punishment) and behavior-oriented measures (e.g., reported parenting practices and stimulation activities), these were based on self-report and therefore may reflect shifts in awareness or perceived norms rather than fully objective changes in behavior. The absence of observational or multi-informant assessments limits the ability to disentangle cognitive or attitudinal change from sustained behavioral change.” (Line 584-590)*

**Reviewers' Comments:**

Reviewer's Responses to Questions

**Comments to the Author**

Reviewer #1:

1. The response rate after the intervention was high at 92.8%. However, 58.1% of participants did not complete the 12-month follow-up. This loss may bias the analysis of the sustainability of the results.

Authors: Thank you for highlighting this concern. We acknowledge that attrition at the 12-month follow-up was substantial and may introduce bias in the interpretation of long-term sustainability effects.

To assess potential attrition bias, we conducted an attrition analysis comparing baseline characteristics of participants who completed the 12-month follow-up with those who did not. We found no statistically significant differences in baseline primary outcomes between completers and non-completers. However, differences were observed in certain demographic characteristics (e.g., caregiver age, gender, employment status), which we now more clearly acknowledge in the Discussion.

Longitudinal analyses were conducted using multilevel mixed-effects models estimated via maximum likelihood under missing-at-random assumptions. Nevertheless, sustained effects should be interpreted cautiously in light of selective retention. We have strengthened the limitation section accordingly.

The added paragraphs include:

*“An attrition analysis was conducted to compare participants who completed the 12-month follow-up (T3) with those who did not. There were no statistically significant differences in baseline primary outcomes between completers and non-completers, including Early Learning and Stimulation (p = 0.063) and Caregiver-Perpetrated Violence (p = 0.984). However, statistically significant differences were observed for certain demographic characteristics, including child age, caregiver age, caregiver gender, and employment status (Appendix Table 14). These findings suggest that follow-up participation may be associated with specific demographic factors, which are further considered in the Discussion.” (Line 448-456)*

*“In addition, at the 12-month follow-up, attrition was associated with certain demographic characteristics. However, completers and non-completers were comparable in baseline primary outcome levels. This reduces the likelihood that sustained effects were driven solely by baseline differences. Nevertheless, long-term findings should be interpreted with consideration of the potential influence of selective retention.” (Line 600-604)*

2. The main outcomes, including caregiver-provided stimulation and violence against children (VAC), rely on self-reported data. This data can be affected by social desirability bias, especially when discussing sensitive topics like violence.

Authors: Thank you for this important observation. As noted in our response to related comments, we have now strengthened the Methods and Discussion sections to clarify that all outcomes were self-reported, describe measures taken to enhance confidentiality, and explicitly acknowledge the potential for under-reporting and the distinction between attitudinal and behavioral outcomes.

*“All outcome measures were collected via caregiver self-report using encrypted online questionnaires administered by trained data collectors via videoconferencing. Participants were assured of confidentiality, and responses were not shared with preschool staff to reduce social desirability bias.” (Line 262-265)*

*“As outcomes relied on caregiver self-report, under-reporting due to social desirability bias cannot be excluded, particularly for sensitive behaviors such as violent discipline. Moreover, although the study included both attitudinal measures (e.g., endorsement of corporal punishment) and behavior-oriented measures (e.g., reported parenting practices and stimulation activities), these were based on self-report and therefore may reflect shifts in awareness or perceived norms rather than fully objective changes in behavior. The absence of observational or multi-informant assessments limits the ability to disentangle cognitive or attitudinal change from sustained behavioral change.” (Line 582-590)*

3. The manuscript states that 21 classes were randomized, but it does not explain the potential differences caused by variations in teachers or facilitators within those classes.

Authors: Thank you for raising this point. In this trial, the intervention was delivered within existing classroom structures. Headteachers facilitated group discussions, and trained social workers provided standardized support across intervention classes to enhance consistency. All facilitators received structured guidance and materials to support fidelity.

To account for between-class variability, multilevel mixed-effects models were used, with participant nested within cluster (class) specified as random effects. This approach accounts for unobserved cluster-level heterogeneity, including potential teacher-level variation.

We have clarified this in the Methods sections.

*“All models specified participant ID nested within cluster (class) as random effects to account for between-class variability, including potential differences in headteachers and facilitation dynamics, and included fixed effects for group (intervention vs control), time (T0, T1, T2, T3; primary inference for between-group effectiveness was restricted to T1), and their interaction (group × time).”* *(Line 298-303)*

4. It is important to compare the participants who completed the 12-month follow-up with those who dropped out. This will help us understand if the "sustained effects" reflect the overall population.

Authors: Thank you for your suggestion. We have now conducted an attrition analysis comparing intervention participants who completed the 12-month follow-up (T3) with those who did not. The findings are reported in the Results section and also presented in Appendix Table 14.

*“An attrition analysis was conducted to compare participants who completed the 12-month follow-up (T3) with those who did not. There were no statistically significant differences in baseline primary outcomes between completers and non-completers, including Early Learning and Stimulation (p = 0.063) and Caregiver-Perpetrated Violence (p = 0.984). However, statistically significant differences were observed for certain demographic characteristics, including child age, caregiver age, caregiver gender, and employment status (Appendix Table 14). These findings suggest that follow-up participation may be associated with specific demographic factors, which are further considered in the Discussion.” (Line 448-456)*

Although statistically significant differences were observed for certain demographic characteristics (e.g., child age, caregiver age, caregiver gender, and employment status), baseline primary outcome levels were comparable between completers and non-completers. We have also acknowledged this pattern in the discussion of limitations.

*“Also, at the 12-month follow-up, attrition was associated with certain demographic characteristics. However, completers and non-completers were comparable in baseline primary outcome levels. This reduces the likelihood that sustained effects were driven solely by baseline differences. Nevertheless, long-term findings should be interpreted with consideration of the potential influence of selective retention.” (Line 600-604)*

5. In the Methodology section, specifically in the Data Management/Storage subsection. It is suggested to refer from the paper: “Risk Assessment for Identifying Threats, Vulnerabilities, and Countermeasures in Cloud Computing “. Since this intervention uses a chatbot and likely relies on cloud storage for caregiver data, citing Addula et al. (2025) adds important context about data safety. The referenced paper highlights weak access controls as a significant threat, accounting for 12% of incidents. This supports the need for robust "deidentified participant data" protocols outlined in the manuscript, along with effective measures such as encryption for sensitive information.

Authors: Thank you very much for this thoughtful suggestion. We agree that data security is an important consideration in digital health interventions. In this study, caregiver data were collected through encrypted online questionnaires and stored on secure, password-protected servers in accordance with institutional data protection policies and ethics approvals. Identifiable information was separated from survey responses, and only de-identified data were used for analysis.

As the focus of the present study was on intervention effectiveness rather than cloud infrastructure risk assessment, we did not include a detailed discussion of cloud security frameworks. However, we have clarified the data protection procedures in the Ethics Statement section to strengthen transparency regarding data safety measures.

*“All data were collected via encrypted online questionnaires and transmitted through secure connections. Survey responses were stored on password-protected servers with access restricted to authorized research personnel. Identifiable information was stored separately from survey data, and only de-identified datasets were used for analysis.” (Line 669-272)*

Thank you again.

6. There is a need more information about the training and consistency of the headteachers and social workers who led the online group sessions.

Authors: Thank you for raising this implementation consideration. We have now expanded the Methods-Intervention Procedures section to provide additional details regarding facilitator training and procedures to support consistency across classes.

*“Regarding training and supervision, headteachers and social workers received a 6-hour structured entry training prior to intervention delivery. Training covered the theoretical foundations of the program, facilitation principles, use of the digital platform, and ethical considerations. Facilitators were provided with a standardized implementation manual, including structured guidance and suggested scripts for group interactions to support consistency across classes. During the intervention period, weekly supervision sessions were conducted to monitor progress and address implementation challenges. Ongoing support was available as needed to ensure procedural consistency and fidelity. Although minor variations in facilitation style were expected in this pragmatic setting, core intervention content was standardized through chatbot-delivered modules and structured discussion themes.” (Line 228-237)*

7. The manuscript should better define the specific "human" elements used to encourage participants to complete the chatbot modules. This will help others replicate the study.

Authors: Thank you for this valuable suggestion. We have now described in greater detail to clarify how the digital and human components were integrated.

*“Given that caregiver participation was central to intervention delivery, human elements were intentionally designed to enhance engagement. These human supports operated across institutional, relational, and motivational levels, while serving four core engagement functions: accountability, social reinforcement, guided reflection, and behavioral reinforcement.*

*At the institutional level, the program was introduced and endorsed by headteachers, who maintained established and trusted relationships with families. Positioning the intervention within routine preschool parenting support services helped legitimize participation, reduce stigma, and normalize engagement within existing school–family structures.*

*At the relational level, weekly or twice-weekly message-based group discussions were facilitated by headteachers and social workers in existing WeChat class groups. These sessions aligned with ongoing chatbot topics and fostered peer exchange, collective participation, and social accountability. Facilitators used structured prompts and suggested scripts from a standardized manual to guide reflection, clarify misunderstandings, and contextualize digital content within caregivers’ everyday parenting experiences.*

*At the motivational level, a child-centered token incentive mechanism was implemented. Children received small developmentally appropriate rewards linked to caregiver engagement with chatbot modules, indirectly reinforcing sustained participation through family-level motivation. In addition, facilitators provided regular reminders and encouragement through established communication channels to prompt timely module completion and maintain engagement momentum.*

*Together, these human elements were integrated to enhance uptake, support adherence, and minimize attrition under real-world implementation conditions.” (Line 206-227)*

8. The document should consistently use terms like "caregiver-perpetrated violence" and "corporal punishment."

Authors: Thank you for your suggestion. We have carefully reviewed the manuscript to ensure consistent terminology throughout. We have standardized the use of “caregiver-perpetrated violence” when referring to behavioral outcomes measured by the ICAST instrument, and “attitudes toward corporal punishment” when referring to the attitudinal measure.

Thank you again.
